# Effects of Core-Inclusive Physical Training on Clubhead Speed in Golfers: A Systematic Review and Meta-Analysis

**DOI:** 10.3390/life16101609

**Published:** 2026-09-25

**Authors:** Hsing-Hui Liu, Long-Huei Lin, Ting-Yu Lin, Wei-Ting Wu, Ke-Vin Chang

**Affiliations:** 1 Department of General Medicine, Changhua Christian Hospital, Changhua 500017, Taiwan; liouhsinghui891023@gmail.com; 2 School of Physical Therapy and Graduate Institute of Rehabilitation Science, College of Medicine, Chang Gung University, Taoyuan 333323, Taiwan; cosx9954022@gmail.com; 3 Department of Physical Medicine and Rehabilitation, Lotung Poh-Ai Hospital, Lo-Hsu Medical Foundation, Inc., Yilan 265501, Taiwan; t840326@icloud.com; 4 Department of Physical Medicine and Rehabilitation, National Taiwan University Hospital, College of Medicine, National Taiwan University, Taipei 100229, Taiwan; 5 Department of Physical Medicine and Rehabilitation, National Taiwan University Hospital, Bei-Hu Branch, Taipei 108206, Taiwan; 6 Center for Regional Anesthesia and Pain Medicine, Wan-Fang Hospital, Taipei Medical University, Taipei 116079, Taiwan

**Keywords:** golf, core muscle training, exercise, physical education, sports

## Abstract

This study aimed to evaluate the effects of core-inclusive physical training, either performed alone or combined with additional physical conditioning, on clubhead speed (CHS) in healthy golfers and to compare golf-specific swing-simulated core training (GSCT) with non-GSCT. Six primary electronic databases (PubMed, Embase, Cochrane CENTRAL, Web of Science, Scopus, PEDro) and Google Scholar were searched from inception to June 2026 for controlled trials. Continuous CHS outcomes were pooled using random-effects models as Hedges’ *g*. Nine trials (220 golfers) were analyzed. Overall core-inclusive physical training showed a small, statistically significant effect on CHS (Hedges’ *g* = 0.290, 95% CI: 0.031 to 0.549, *p* = 0.028; *I*^2^ = 0.000%). Subgroup analysis indicated that interventions incorporating GSCT showed a numerically larger effect (Hedges’ *g* = 0.451, 95% CI: 0.073 to 0.830, *p* = 0.020) than non-GSCT (Hedges’ *g* = 0.148, 95% CI: −0.208 to 0.503, *p* = 0.416). However, meta-regression for between-subgroup differences did not reach statistical significance (*Q* = 1.31, *p* = 0.252). Certainty of evidence according to the GRADE framework was rated as low for overall CHS and very low for the GSCT subgroup training. In conclusion, conditioning practitioners may implement comprehensive core-inclusive physical training to enhance CHS, while considering GSCT as a promising yet exploratory strategy pending confirmation from larger randomized trials.

## 1. Introduction

Golf is a globally popular sport played by more than 60 million people worldwide, ranging from elite professional athletes to recreational participants [1]. Regardless of athletic level, the primary goal of golf is to complete the course in the fewest possible strokes [2]. Clubhead speed (CHS) is one of the most commonly used indicators of golf performance and has been shown to improve following physical training interventions [3]. In addition, CHS has been shown to be strongly associated with golfers’ playing ability, commonly quantified using the Handicap Index, which is calculated from the average of the eight lowest score differentials from a player’s most recent 20 rounds, with lower values indicating superior playing ability [4]. Fradkin et al. [5] reported a strong correlation between CHS and handicap, demonstrating that golfers with lower handicaps exhibit greater CHS. Similarly, Brown et al. [6] found a significant correlation between handicap and CHS, indicating that better golfers generate higher CHS. Although CHS is a critical determinant of driving distance, golf performance is multifaceted, also depending heavily on shot accuracy, approach play, and putting proficiency [7].

To optimize CHS, previous studies have investigated various physical training modalities, including resistance training, plyometrics, and flexibility exercises [8,9]. Recent evidence has demonstrated a strong positive association between peak trunk rotational power and CHS in elite golfers, highlighting the critical role of the core musculature during the high-speed golf swing [10]. Anatomically, the core musculature comprises the axial skeleton and attached soft tissues, primarily the rectus abdominis, transversus abdominis, internal and external obliques, lumbar multifidus, and erector spinae, serving as a critical hub in the rotational kinetic chain [11]. The underlying biomechanical mechanism relies on these muscle groups to establish proximal stability to facilitate distal mobility of the extremities, thereby increasing spinal stiffness and facilitating proximal-to-distal energy transfer during kinetic transfer [12].

Although current systematic reviews have shown that physical training improves CHS, they have not specifically examined the effects of training programs involving core muscle training [8,9]. Therefore, this systematic review and meta-analysis aimed to evaluate the effects of core-inclusive physical training, either performed alone or combined with additional physical conditioning on CHS. To further assess the training effects, subgroup analyses will be conducted based on training specificity, specifically distinguishing between golf-specific swing-simulated core training (GSCT) and non-GSCT regimens. Additionally, exploratory meta-regression analyses were conducted to examine whether sex, age, and intervention duration acted as potential effect modifiers.

## 2. Materials and Methods

### 2.1. Study Design and Registration

This systematic review and meta-analysis was conducted in accordance with the PRISMA 2020 reporting guidelines [13], and the reporting checklist is available in Table S1. The study protocol was prospectively registered with INPLASY under the registration number (INPLASY202680072). Ethical approval was waived because this study exclusively utilized published data from previous trials.

### 2.2. Literature Search Strategy

Two independent researchers conducted a comprehensive literature search across PubMed, Embase, Cochrane CENTRAL, Web of Science, Scopus, PEDro and Google Scholar from their inception to 30 June 2026. For Google Scholar, given its dynamic ranking algorithm and large volume of records, search results were sorted by relevance, and only the first 200 citations were screened as an a priori stopping rule, consistent with established methodological recommendations for systematic reviews [14]. We used Boolean operators to combine search terms related to the population and interventions, specifically (“Golf” OR “Golfer”) AND (“Core training” OR “Core stability” OR “Core exercise” OR “Trunk training” OR “Trunk stability” OR “Lumbopelvic” OR “Spinal stabilization” OR “Plyometric” OR “Resistance training” OR “Strength training” OR “Functional training” OR “Corrective exercise” OR “Weight ball” OR “Medicine ball”). The explicit, reproducible search strings for all databases are provided in the Supplementary Material (Table S2). Furthermore, reference lists of retrieved full-text articles and systematic reviews were manually searched. Any screening disagreements were resolved through consensus or arbitration by a third senior researcher.

### 2.3. Eligibility Criteria

The inclusion criteria were defined according to the PICO framework: (1) population: healthy golfers of any skill level, age, or sex; (2) intervention: structured core-inclusive physical training programs, defined as physical conditioning protocols incorporating targeted core muscle exercises, delivered either as an isolated core regimen or within a multimodal conditioning program combined with upper- and/or lower-extremity conditioning. The term “core” was defined as an anatomical box comprising the rectus abdominis anteriorly; the internal and external oblique muscles laterally; the erector spinae, lumbar multifidus, and quadratus lumborum posteriorly; the diaphragm superiorly; and the pelvic floor and iliopsoas inferiorly [11]; (3) comparison: traditional training, active control, or placebo control; and (4) outcome: quantitative assessment of CHS. Studies were excluded if they were observational studies, case reports, or cohort studies; included participants with acute musculoskeletal injuries; lacked an appropriate control group; or reported insufficient data for meta-analysis. No language restrictions were initially applied. Unpublished dissertations, conference proceedings, and abstracts without complete data were excluded.

### 2.4. Methodological Quality Appraisal

The methodological quality of the included articles was assessed using the Cochrane Risk of Bias 2 (RoB 2) tool. This tool evaluates bias across five domains: the randomization process, deviations from intended interventions, missing outcome data, measurement of the outcome, and selection of the reported result. For the non-randomized controlled trial, the Risk of Bias in Non-randomized Studies—of Interventions (ROBINS-I) tool was employed, which evaluates bias across seven domains: confounding, selection of participants, classification of interventions, deviations from intended interventions, missing data, measurement of outcomes, and selection of reported results. Two reviewers independently performed all quality appraisals, and disagreements were resolved through consensus or arbitration by a third senior author.

Furthermore, the overall certainty of evidence for the CHS was assessed using the Grading of Recommendations Assessment, Development, and Evaluation (GRADE) framework [15]. The evidence was categorized into high, moderate, low, or very low quality based on five downgrading domains: study limitations, inconsistency of results, indirectness of evidence, imprecision, and publication bias.

### 2.5. Outcome Measurement

The outcome was the change in CHS following the intervention. CHS is typically measured in miles per hour (mph) or meters per second (m/s) using launch monitors (e.g., radar or optical systems) or 3D motion analysis. It serves as a key biomechanical metric that reflects a golfer’s swing efficiency and force generation, directly influencing the potential distance of the golf shot.

### 2.6. Data Extraction and Management

Data were independently extracted by two reviewers, encompassing study characteristics, participant demographics, physical training and control protocols, and outcome values. In cases of multiple follow-up measurements, data extraction was strictly standardized to the final post-intervention time point. For studies reporting CHS across multiple club types, data from the driver were prioritized to evaluate maximal swing velocity, followed hierarchically by longer irons (e.g., 5- or 6-iron over 7-iron).

All CHS measurements were uniformly converted to m/s. Standard errors were converted to standard deviations (SDs) using established formulas [16]. To account for potential baseline differences, the effect size for each study was calculated based on the between-group difference in pre-to-post changes in CHS (i.e., the change from baseline to post-intervention in the intervention group compared with the corresponding change in the control group), rather than on post-intervention values alone. When standard deviations of change scores were unavailable, they were derived following the Cochrane Handbook [16] with an assumed correlation coefficient of r=0.5 between baseline and post-intervention values. For studies reporting baseline values and percentage changes, post-intervention means were reconstructed accordingly. In instances where post-intervention SDs were unavailable, baseline SDs were substituted under the assumption of equal variance across the intervention. Detailed information concerning data extraction and numerical values is summarized in Table S3.

### 2.7. Statistical Analysis

Given the anticipated clinical and methodological heterogeneity across diverse physical training protocols, a random-effects model was used for the meta-analysis [17]. Although CHS measurements were converted to m/s, included trials were anticipated to utilize varied golf clubs (e.g., driver vs. irons), which could generate substantial variance in baseline velocities and measurement scales. Between-study variance $\tau^{2}$ was estimated using the DerSimonian and Laird method. Between-study heterogeneity was quantified via the Cochran *Q* test and *I*^2^ statistic. Standardized mean difference (Hedges’ *g*) with small-sample bias correction was therefore preferred over raw mean difference to standardize treatment effects relative to study-specific variability [18]. Continuous outcomes were pooled as Hedges’ g with 95% confidence intervals (CIs) using Comprehensive Meta-Analysis software (version 4; Biostat, Englewood, NJ, USA). Effect sizes were classified as small (0.2), moderate (0.5), or large (0.8) [18]. Heterogeneity was evaluated via the Cochran Q statistic and the *I^2^* index, with *I^2^* values of 25%, 50%, and 75% representing low, moderate, and high heterogeneity, respectively [19]. A two-tailed *p*-value < 0.05 was considered statistically significant.

To explore potential sources of heterogeneity, a prespecified subgroup analysis was conducted according to training specificity by categorizing interventions into GSCT versus non-GSCT. To qualify as GSCT, an intervention was required to satisfy the following minimum operational criteria: (1) incorporation of multi-planar or dynamic diagonal-rotational trunk movements (e.g., medicine ball rotational throws, cable chops, or simulated golf-swing resistance exercises) rather than solely static, uni-planar, or non-rotational core stabilization exercises; and (2) execution of these rotational exercises targeting the stretch-shortening cycle and rapid proximal-to-distal force transmission across the kinetic chain in a trajectory directly mimicking the kinematic phases of the golf swing. In contrast, interventions focusing primarily on general isometric holds, linear trunk flexion/extension, dynamic stretching, or general lower-extremity/squat patterns without rotational swing simulation were categorized as non-GSCT. Two reviewers independently classified the interventions, and any disagreements were resolved by consultation with the corresponding author. Additionally, subgroup analyses were conducted to determine whether combining core muscle training with upper- and/or lower-extremity training (e.g., plyometric or resistance training) influenced the intervention effect. On the other hand, because this review included both randomized and non-randomized controlled studies, an additional subgroup analysis based on methodological quality was conducted to determine whether study quality influenced the overall results. To formally assess differences in intervention effects between subgroup categories, categorical meta-regression analyses were additionally performed. Between-category differences were evaluated using the Q statistic for the moderator (Q-model), with the corresponding *p* value reported.

The other exploratory meta-regression analyses were conducted to determine whether continuous variables (intervention length, training frequency, proportion of male participants, and participant age) acted as significant modifiers of the overall treatment effect, as these factors are widely recognized in the literature as critical determinants of physiological adaptations and performance outcomes in sports interventions [20,21]. Sensitivity analyses using the leave-one-out method were performed to confirm the robustness of the findings [22]. To assess potential publication bias, we adhered to the methodological recommendations outlined in the Cochrane Handbook for Systematic Reviews of Interventions. Funnel plot asymmetry was visually assessed, with Egger’s test used as a confirmatory analysis [23].

## 3. Results

### 3.1. Study Identification and Selection

The comprehensive literature search and systematic screening process were executed in accordance with the PRISMA guidelines, as delineated in the PRISMA flowchart (Figure 1). The initial multi-database electronic search yielded a total of 840 records. Following the removal of duplicate citations and the exclusion of non-relevant articles during the initial title and abstract screening phase, 15 full-text articles were rigorously assessed for eligibility. Ultimately, eight randomized controlled trials and one non-randomized controlled trial satisfied all predefined selection criteria and were incorporated into the quantitative meta-analysis. The explicit reasons for study exclusion at the definitive full-text review stage are systematically cataloged in Supplementary Table S4.

### 3.2. Characteristics of Included Studies

The nine eligible trials encompassed an aggregate sample size of 220 healthy golfers, with a mean age of 37.21 years, of whom 65.5% (*n* = 144) were male, 26.4% (*n* = 58) were female, and 8.2% (*n* = 18) were of unspecified sex. The physical training protocols ranged in duration from 6 to 12 weeks. The cohorts primarily consisted of amateur, recreational, and club-level golfers, although specific trials investigated highly trained populations including competitive elite or collegiate players. The detailed baseline demographics and performance characteristics of the synthesized training cohorts are summarized in the study characteristics table (Table 1).

The core muscle training program designs varied across the included literature, allowing for the evaluation of isolated versus multi-modal conditioning protocols. Participant cohorts comprised recreational, club-level, collegiate, and elite competitive golfers. Only one trial evaluated an isolated core training regimen [30], whereas the remaining eight implemented multimodal conditioning combining core training with upper- and/or lower-extremity exercises [24,25,26,27,28,29,31,32]. Regarding training specificity, six trials incorporated dynamic GSCT drills replicating swing kinematics [24,25,27,28,29,30], whereas three implemented non-GSCT core protocols [26,31,32] (Table 2). Control conditions consisted of active golf practice or sham exercise programs.

### 3.3. Methodological Quality Assessment

For the eight RCTs evaluated via RoB 2, one trial (12.5%) demonstrated a low overall risk of bias, six (75.0%) raised some concerns, and one (12.5%) was rated as high risk due to substantial attrition (Table 3, Figure S1). Key concerns stemmed primarily from incomplete reporting of allocation concealment and sequence generation. For one non-randomized trial [28], evaluation via ROBINS-I indicated a moderate overall risk of bias (Table 4), driven mainly by potential residual confounding from non-random matched allocation and absence of a pre-registered analysis plan.

### 3.4. Effect of Core-Inclusive Physical Training on CHS

Overall core-inclusive physical training demonstrated a small, statistically significant effect on CHS compared with control conditions (Hedges’ *g* = 0.290, 95% CI: 0.031 to 0.549, *p* = 0.028, *I*^2^ = 0.000%; Figure 2). The DerSimonian and Laird random-effects model yielded a between-study variance estimate of zero ($\tau^{2}$ = 0.000, τ = 0.000; Cochran’s Q = 5.910, df = 8, *p* = 0.657). In a prespecified sensitivity analysis excluding the non-randomized trial [28], the pooled estimate remained statistically significant (8 trials; Hedges’ *g* = 0.280, 95% CI: 0.010 to 0.550, *p* = 0.042; *I^2^* = 0.000; Figure S2). Sequential leave-one-out sensitivity analyses showed that the pooled effect estimate maintained a similar trend (Figure S3).

Regarding training specificity, core interventions incorporating GSCT demonstrated a significant positive effect on CHS (sixs trials; Hedges’ *g* = 0.451, 95% CI: 0.073 to 0.830, *p* = 0.020, *I*^2^ = 0.000%) (Figure 3), whereas non-GSCT core regimens did not yield statistically significant improvements (three trials; Hedges’ *g* = 0.148, 95% CI: −0.208 to 0.503, *p* = 0.415, *I*^2^ = 0.000%). Categorical meta-regression revealed no statistically significant difference in intervention effects between the GSCT and non-GSCT subgroups (Q = 1.310, df = 1, *p* = 0.252; Figure S4). Furthermore, given that the trial by Choi et al. exhibited an exceptionally large effect size (Hedges’ *g* = 1.146), a subgroup-specific leave-one-out analysis was performed and showed a similar trend to the primary analysis. This analysis rendered the GSCT effect statistically non-significant (Figure S5) [30].

**Figure 2:**
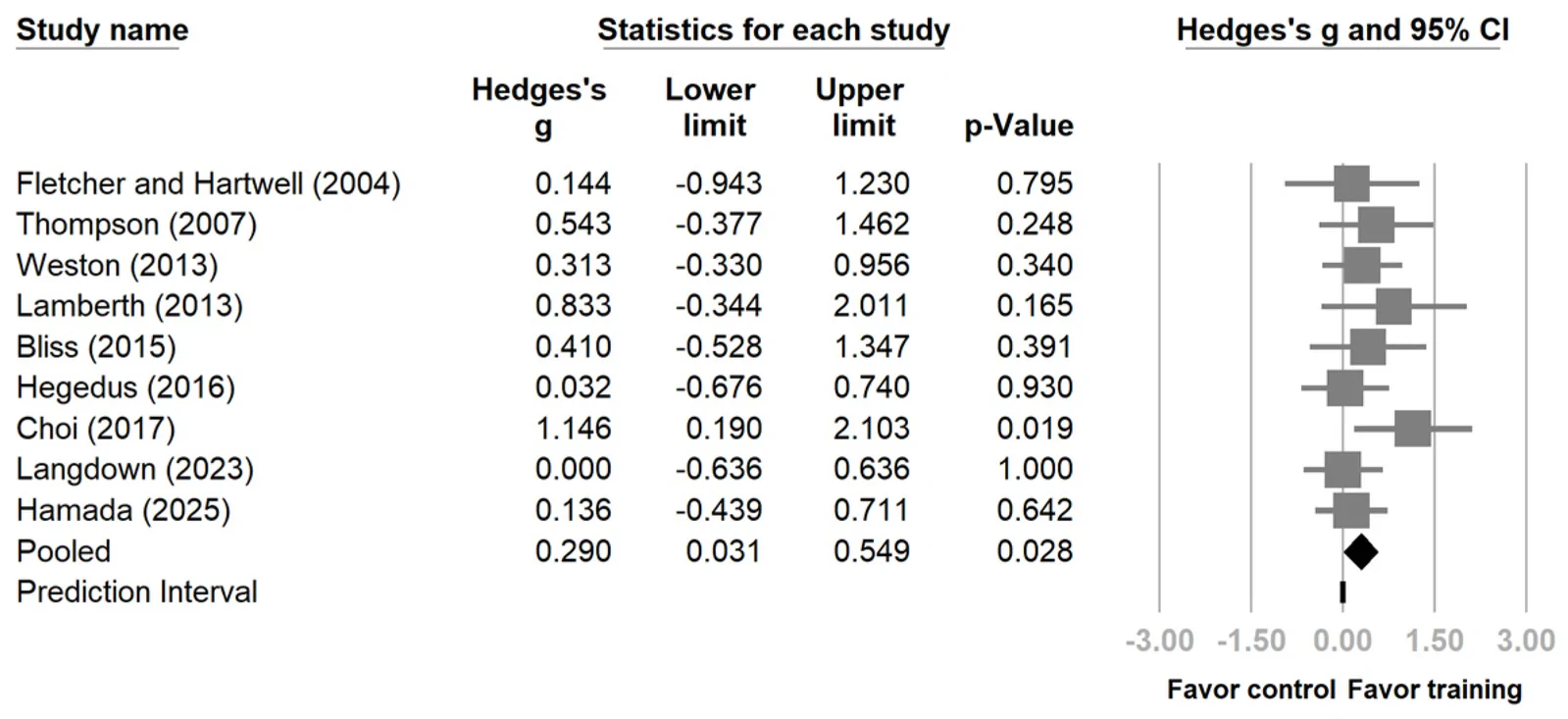
Forest plot of the effects of core-inclusive physical training on CHS [24,25,26,27,28,29,30,31,32].

**Table 2:** Summary of the core-inclusive physical training administered in the study treatment arms of the retrieved trials.

First Author and Year	Training Regimen (Duration, Frequency and Volume)	Core Focus Classification	Specificity ^1^	Core-Inclusive Intervention Details (Focus and Description)	Control Description	Outcomes
Fletcher (2004) [24]	8 weeks; 2 sessions/week, 90 min/session; 3 sets × 6–8 reps (weights), 3 sets × 8 reps (plyometrics)	Multimodal (core + resistance + plyometrics)	GSCT	Combined weights and plyometrics: 9 resistance exercises (e.g., bench press, squats, lunges) and 4 explosive MB (3 kg) exercises simulating swing with ball release to maximize sequential acceleration.	Normal training (mainly aerobic exercise and light machine weights)	CHS and driving distance
Thompson (2007) [25]	8 weeks; 3 sessions/week, 90 min/session; Core, balance, and strength circuits	Multimodal (core + balance + circuit resistance)	GSCT	Progressive functional training: Weeks 1–6: core stabilization, static/dynamic balance, and resistance circuit (e.g., floor bridging, single-leg balance, wall push-ups). Weeks 7–8: progressed to MB rotations, wood chops, and simulated cable swings.	Continue current level of physical activity and normal golf practice	CHS and SFT variables (chair stand, arm curl, step test, etc.)
Weston (2013) [26]	8 weeks; 3 sessions/week; 8 exercises with 10–15 s holds	Multimodal (core + lower)	Non-GSCT	Isolated core training: Neutral spine isometric focus. Weeks 1–4: 8 basic core exercises (e.g., squat, prone bridge, lunge) with 10 s holds. Weeks 5–8: progressed by adding limb movements and lengthening holds to 15 s.	Continue normal levels of physical activity including playing golf	CHS, ball backspin, ball sidespin, and isometric flexor endurance test
Lamberth (2013) [27]	6 weeks; 3 sessions/week; 3 sets × 8–25 reps	Multimodal (core + general resistance)	GSCT	Strength and functional training: 21 general resistance and functional exercises targeting upper/lower body and core (e.g., back extension, leg press, bench press, DB rows, standing cable twist, cable woodchop).	Continue regular golf routine and practice (≥4 days/week) without strength training	CHS, 1RM bench press, 1RM leg press, vertical jump, and sit-and-reach flexibility
Bliss (2015) [28]	8 weeks; 2 sessions/week; 10 min warm-up + main session (3–4 sets × 4–6 reps)	Multimodal (core + plyometrics)	GSCT	Plyometric training: Weeks 2–5: 3 sets of vertical jumps, box jumps, and MB (5 kg) rotational throws. Weeks 7–10: progressed to 4 sets of kneeling rotational throws, lateral hops, and simulated golf swing throws with ball release.	Continue normal golf-specific training and competition play	Hit distance, CHS, CD, BS, standing vertical jump, broad jump, and kneeling chest/rotational throw
Hegedus (2016) [29]	10 weeks; 3 sessions/week; 9 exercises × 3 sets × 10 reps	Multimodal (core + unstable surface resistance)	GSCT	GSRT: Multi-planar strength and dynamic balance exercises on unstable surfaces (e.g., Bosu ball back extensions, standing diagonal chops, and lateral plyometrics).	TRAD (uni-directional, machine-based, stable support)	Driver and 7-iron CHS, driver and 7-iron shot distance, body composition, BMD, broad jump, weighted ball throw, and rotation ROM
Choi (2017) [30]	8 weeks; before golf practice, 20 min/session; 4–5 throws/minute	Isolated core (rotational power)	GSCT	Rotational weight ball throws: Preparatory rotational conditioning. Swing trajectory throws from waist to opposite shoulder with weight shifting. Weeks 1–4: 3 kg ball. Weeks 5–8: 5 kg ball thrown to maximize rotational acceleration.	Practice golf for the same time without weight ball throw training	RBET, WSLB, CHS and DD
Langdown (2023) [31]	8 weeks; 3–4 sessions/week; 15 min dynamic warm-up + main session	Multimodal (core + corrective/mobility)	Non-GSCT	Strength and corrective exercise: Targeted gluteal strength, thoracic mobility, and lumbopelvic stability designed to correct overhead deep squat patterns.	Continue normal practice and playing routine	3D 6-iron swing kinematics, CHS, BS, and OHS variables (torso, thigh, shin, and arm angles)
Hamada (2025) [32]	12 weeks; before golf practice, 2 sets; 6 exercises × 5–10 reps	Multimodal (core + dynamic stretch/LBP Prevention)	Non-GSCT	GLEP: Dynamic stretching targeting lead hip external rotation and spinal mobility (e.g., hip/trunk rotation, forward lunges with rotation, and squat jumps) to increase trunk stiffness.	Sham program (dynamic stretches excluding hip ER and spine rotation)	CHS, BS, CD, spin rate, launch angle, hip/spine ROM, finger floor distance, HipSIT, and side bridge test

Abbreviations: BMD, bone mineral density; BS, ball speed; CHS, clubhead speed; CD, carry distance; DD, driving distance; ER, external rotation; GLEP, golfers’ low back pain exercise prevention program; GSCT, golf-specific swing-simulated core training; MB, medicine ball; OHS, overhead squat; RBET, rotational back extension torque; SFT, Senior Fitness Test; TRAD, traditional resistance training; WSLB, weight shifting of lower body. ^1^ GSCT indicates core exercises integrating dynamic rotational velocity movements designed to replicate actual swing trajectories; Extremity Integration defines whether core muscle training was performed in isolation or combined with additional extremity conditioning, targeting either the lower limbs only or both the upper and lower body.

**Table 3:** Risk of bias assessment for included randomized controlled trials using the revised Cochrane Risk-of-Bias Tool (RoB 2).

First Author (Year)	Randomization Process ^1^	Intervention Adherence ^4^	Missing Outcome Data	Outcome Measurement ^7^	Selective Reporting ^8^	Overall RoB
Fletcher 2004 [24]	S ^1^	L ^2^	L	L	S	S
Thompson 2007 [25]	S ^1^	L ^2^	L	L	S	S
Weston 2013 [26]	S ^1^	L ^2^	L	L	S	S
Lamberth 2013 [27]	S ^1^	L ^2^	L	L	S	S
Hegedus 2016 [29]	S ^3^	H ^5^	H ^5^	L	S	H
Choi 2017 [30]	S ^1^	L ^2^	L	L	S	S
Langdown 2023 [31]	S ^1^	S ^6^	L	L	S	S
Hamada 2025 [32]	L	L	L	L	L	L

H, high risk of bias; L, low risk of bias; RoB, risk of bias; S, some risk of bias. ^1^ Randomized allocation was stated with balanced baseline characteristics. However, owing to incomplete reporting of allocation concealment and exact sequence generation details. ^2^ In exercise trials, blinding is inherently impossible. Interventions were delivered as planned in real-world sports contexts without unapproved contextual deviations; they are rated as low risk. ^3^ Significant baseline imbalances in key prognostic factors raise some concerns despite adequate allocation concealment. ^4^ Blinding is impossible in exercise trials. Rated low risk because protocol deviations reflected real-world contexts and all participants were analyzed appropriately. ^5^ High intervention-related attrition (~35%) and naïve per-protocol analysis without statistical correction indicate high risk in adherence and missing data domains. ^6^ Excluding minor control group dropouts (~10%) from the final analysis constitutes a naïve per-protocol analysis, raising some concerns for adherence. ^7^ Measurement risk is low because golf performance was evaluated using objective, automated instruments (e.g., launch monitors). ^8^ Trials lacking a prospectively published trial registry or Statistical Analysis Plan (SAP) prior to unblinded analysis are rated as Some concerns.

**Table 4:** Risk of bias assessment for non-randomized studies of interventions using the ROBINS-I tool.

First Author (Year)	D1	D2	D3	D4	D5	D6	D7	Overall RoB
Bliss 2015 [28]	M ^1^	L	L	L	L	L	M ^2^	M

L, low risk of bias; M, moderate risk of bias. ^1^ Participants were non-randomly allocated into two groups matched for age and handicap. Although baseline CHS and BCD showed no statistically significant group differences, matching on a small sample without controlling for unmeasured physical maturity or baseline power covariates leaves residual confounding risk. ^2^ The study lacked a pre-registered prospective trial protocol or analysis plan (SAP).

**Figure 3:**
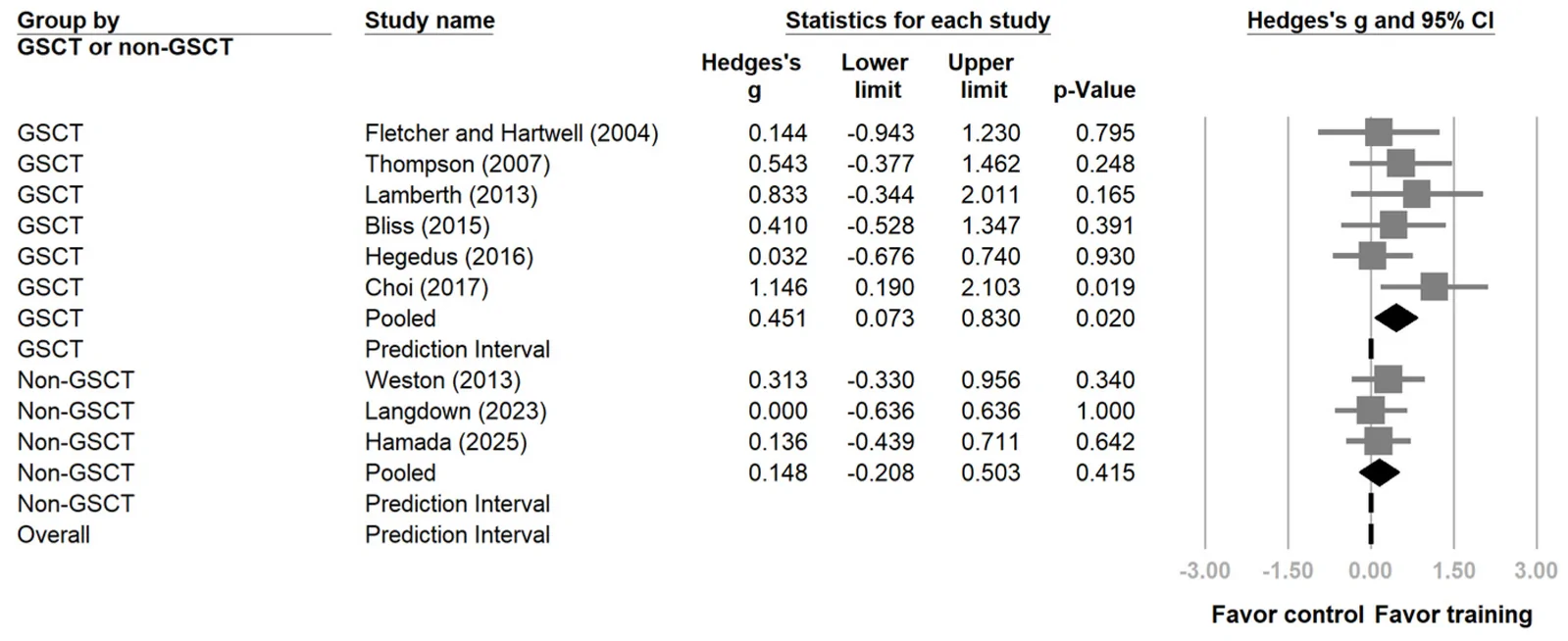
Subgroup analysis forest plot assessing the effects of core-inclusive physical training on CHS categorized by GSCT [24,25,27,28,29,30] or non-GSCT [26,31,32].

Regarding the integration of extremity conditioning, core training combined with lower-extremity conditioning (5 trials; Hedges’ *g* = 0.154, 95% CI: −0.147 to 0.455, *p* = 0.316, *I*^2^ = 0.000%) and core training combined with both upper- and lower-extremity conditioning (3 trials; Hedges’ *g* = 0.496, 95% CI: −0.107 to 1.099, *p* = 0.107, *I*^2^ = 0.000%) did not elicit significant changes (Figure 4). Consistent with the extensive overlap of the 95% CIs across these subgroups, categorical meta-regression confirmed that the inclusion of upper- and/or lower-extremity training did not significantly moderate the overall efficacy of core-inclusive protocols on CHS (*Q* = 4.320, *df* = 2, *p* = 0.116; Figure S6).

When stratified by methodological quality, the pooled effect sizes were Hedges’ *g* = 0.169 (95% CI: −0.396 to 0.734, *p* = 0.557, *I*^2^ = 0.000%) for the high-risk subgroup (2 trial), Hedges’ *g* = 0.136 (95% CI: −0.439 to 0.711, *p* = 0.642, *I*^2^ = 0.000%) for the low-risk subgroup (1 trial), and Hedges’ *g* = 0.387 (95% CI: 0.048 to 0.725, *p* = 0.025, *I*^2^ = 0.000%) for the some-concerns subgroup (7 trials) (Figure 5). Categorical meta-regression indicated no statistically detectable difference across risk-of-bias categories (Q = 0.76, df = 2, *p* = 0.683; Figure S7). However, this non-significant result must be interpreted with extreme caution due to the very limited statistical power inherent in comparing subgroups that contain only a single trial.

Exploratory meta-regression analyses revealed that none of the continuous moderators acted as significant modifiers of the primary treatment effect, including training duration (9 trials, *Z* = −1.08, *p* = 0.282; Figure S8), training frequency (6 trials, *Z* = −0.35, *p* = 0.726; Figure S9), mean participant age (9 trials, *Z* = −0.03, *p* = 0.974; Figure S10, and the proportion of male participants (8 trials, *Z* = 0.82, *p* = 0.411; Figure S11). However, these non-significant findings reflect restricted statistical power inherent in meta-regressions with fewer than ten trials rather than definitive proof of absent moderation.

### 3.5. Publication Bias

Funnel plot inspection revealed mild visual asymmetry (Figure S12), and Egger’s linear regression test yielded a borderline result (*p* = 0.057).

### 3.6. Certainty of Evidence

The overall certainty of evidence for core-inclusive physical training on CHS was rated as low (Table S5). The initial high certainty from RCTs was downgraded by two levels due to study limitations and imprecision. For the GSCT subgroup, certainty was rated as very low, downgraded for risk of bias and severe imprecision driven by single-study dominance (Table S5).

## 4. Discussion

The present meta-analysis found a small but imprecisely estimated overall effect of core-inclusive physical training on CHS in golfers. Although interventions incorporating GSCT showed a statistically significant within-subgroup effect and a numerically larger effect estimate than non-GSCT, categorical meta-regression did not detect a statistically significant difference between the two subgroups. Therefore, while the findings suggest a potentially favorable effect of GSCT, the present evidence does not establish an additional benefit of GSCT over non-GSCT. Similarly, no statistically significant between-subgroup difference was detected according to the inclusion of extremity conditioning. Furthermore, exploratory meta-regressions identified no statistically detectable moderating associations for training duration, frequency, age, or sex distribution. These findings suggest that incorporating GSCT may offer a favorable trend for enhancing CHS, though definitive conclusions warrant further verification in adequately powered trials.

To the best of our knowledge, this may be the first meta-analysis to specifically examine whether incorporating golf-specific core training into physical training programs provides additional benefits for improving CHS in golfers. Previous systematic reviews have primarily focused on the overall effects of physical training or resistance training without considering the role of core training. For example, Robinson et al. demonstrated that physical training significantly improved CHS in female golfers [8]. However, they did not perform subgroup analyses according to training modality. Similarly, Uthoff et al. systematically reviewed resistance training interventions but did not isolate core training as a distinct intervention category [9]. Furthermore, previous meta-analyses investigating core training have generally pooled sport-specific outcomes across different sports, such as throwing velocity, golf driving distance, and swimming or rowing performance, into a single outcome [33,34]. Because the biomechanical demands differ substantially among sports, these pooled analyses may obscure sport-specific training adaptations. Therefore, the present study addresses an important knowledge gap by providing the first sport-specific quantitative synthesis comparing physical training with and without golf-specific core training for improving CHS in golfers.

The numerically favorable trend observed with GSCT may be explained by plausible biomechanical hypotheses. The core musculature serves as an anatomical hub in the kinetic chain, facilitating a sequential proximal-to-distal transfer of energy [35]. Dynamic electromyography investigations demonstrate marked activation of trunk musculature during the swing phase, with the obliques contributing to torso rotation and the erector spinae and gluteal muscles co-contracting to provide dynamic lumbopelvic stability [36]. Biomechanically, this muscular coordination is inferred to enhance proximal stability, potentially facilitating efficient energy transfer across spinal segments without substantial kinematic breakdown [37]. Furthermore, the golf swing relies on a rapid rotational stretch-shortening cycle, where pelvis-thorax angular separation (the “X-factor stretch”) stretches the trunk musculature prior to downswing acceleration [38]. Highly skilled players demonstrate greater X-factor separation and rapid trunk deceleration before impact compared to recreational golfers [38]. Crucially, this rotational sequence must be executed within a highly restricted time frame, given that the downswing typically lasts less than 300 milliseconds [39]. Consequently, GSCT may address this requirement by replicating the movement kinematics and explosive velocities of the actual golf swing, which may facilitate the transfer of gains in rotational power to CHS [40]. In elite male golfers, trunk rotation peak power has been reported to be strongly associated with CHS (r = 0.89) [10]. Although this cross-sectional association does not establish causality, it suggests a potential relationship between trunk power and swing performance.

On the other hand, no statistically significant between-subgroup difference was detected with respect to the inclusion of upper- and/or lower-extremity training. Crucially, this finding must be interpreted with extreme caution because the isolated core training subgroup comprised only a single investigation [30]. Previous meta-analytic evidence has shown that CHS is moderately associated with both upper- and lower-body strength (z_r_ = 0.47 and 0.48, respectively), whereas stronger associations have been observed for measures of jumping performance, including jump displacement (z_r_ = 0.53), jump impulse (z_r_ = 0.82), and jump peak power (z_r_ = 0.66) [4]. Moreover, previous studies have commonly used jumping performance as an indicator of lower-extremity explosive power [41]. However, most studies included in the present meta-analysis primarily incorporated conventional upper- and/or lower-extremity resistance exercises, with only Bliss et al. including vertical jumps [28]. Therefore, the lack of an observed moderating benefit from extremity conditioning likely reflects both insufficient statistical power from a limited number of trials and the scarce inclusion of explosive or plyometric training across the evaluated programs, rather than true equivalence between isolated and multimodal regimens.

Despite the novelty of this review, several limitations should be acknowledged. First, the overall evidence base was small (nine trials), including one non-randomized study [28], which may limit the precision and generalizability of the pooled estimates. Future studies with larger sample sizes and well-designed randomized controlled trials are needed to strengthen the evidence and confirm the present findings. Second, although *I*^2^ was 0.000%, substantial clinical and methodological heterogeneity remained across the included studies. We explored potential sources of heterogeneity through subgroup and meta-regression analyses. However, other differences remained, including the use of different golf clubs for CHS assessment, variations in control conditions and competitive level. Therefore, the low *I*^2^ should not be interpreted as evidence of genuine homogeneity. Future studies should adopt more standardized assessment methods, intervention protocols, and control conditions to improve comparability across studies. Third, the lack of long-term follow-up and scarce reporting of ball speed or carry distance restricted the comprehensive evaluation of performance retention. Fourth, the use of different golf club types for CHS assessment may represent a source of methodological heterogeneity. However, the limited number of studies within each club category and incomplete reporting of club type in some studies precluded a meaningful subgroup analysis. Future studies should clearly report the club type used for CHS assessment and adopt more standardized measurement protocols to facilitate comparisons across studies. Fifth, the meta-regression analyses were exploratory because only a small number of studies were available for each moderator. Consequently, these analyses had limited statistical power and potentially unstable coefficient estimates. Sixth, because only nine studies were included, both visual assessment of funnel plot asymmetry and Egger’s test have limited reliability and statistical power. Accordingly, these findings should be interpreted cautiously and do not exclude the possibility of publication bias or small-study effects. Seventh, given the small number of included studies, future meta-analyses should consider using the Hartung–Knapp adjustment to provide more reliable CIs and account for uncertainty in random-effects models. Eighth, although all subgroup and meta-regression analyses were prespecified in the registered protocol, the multiple secondary analyses conducted with a limited number of included studies may increase the probability of chance findings. Therefore, particularly marginally significant findings should be interpreted cautiously and require confirmation in future studies with a larger evidence base. Finally, the GSCT subgroup included only six studies, and only one study evaluated isolated core training alone, while the remaining trials combined core training with extremity conditioning. Therefore, the independent effect of core training and the apparent advantage of GSCT remain preliminary and require confirmation in larger randomized trials.

## 5. Conclusions

Core-inclusive physical training offers a modest overall benefit for enhancing maximal CHS in healthy golfers. Although GSCT showed a favorable numerical trend, categorical meta-regression did not identify a statistically significant difference between GSCT and non-GSCT, and the certainty of evidence for the GSCT subgroup was very low. Therefore, the apparent advantage of GSCT should be considered preliminary. Core-inclusive physical training may be considered as part of golf conditioning programs, whereas the additional benefit of incorporating GSCT requires confirmation in larger, well-designed randomized controlled trials. From a practical perspective, coaches and practitioners may consider GSCT as one component of golf conditioning programs, pending confirmation in larger comparative trials.

## Figures and Tables

**Figure 1:**
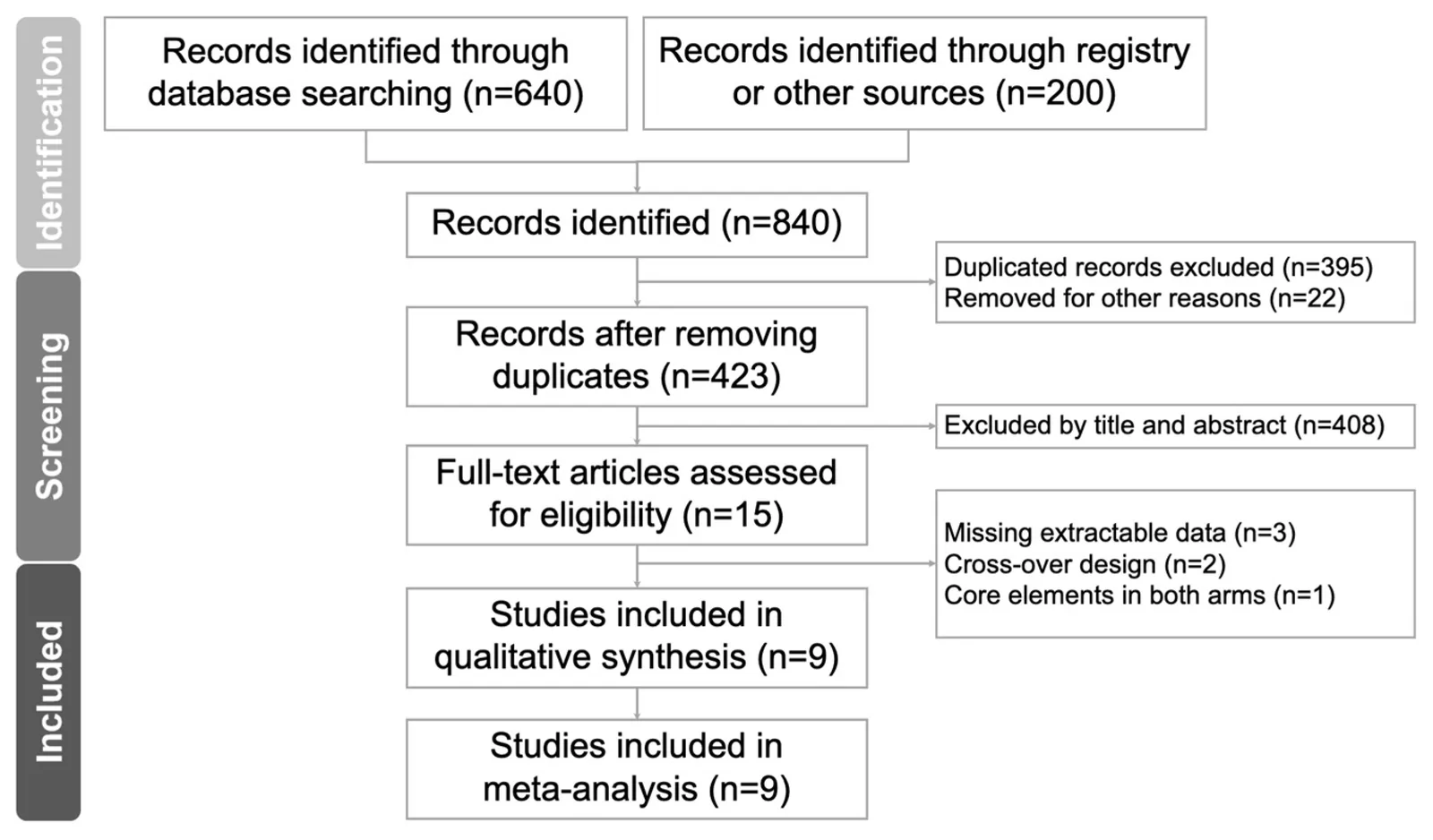
PRISMA 2020 flowchart of current meta-analysis.

**Figure 4:**
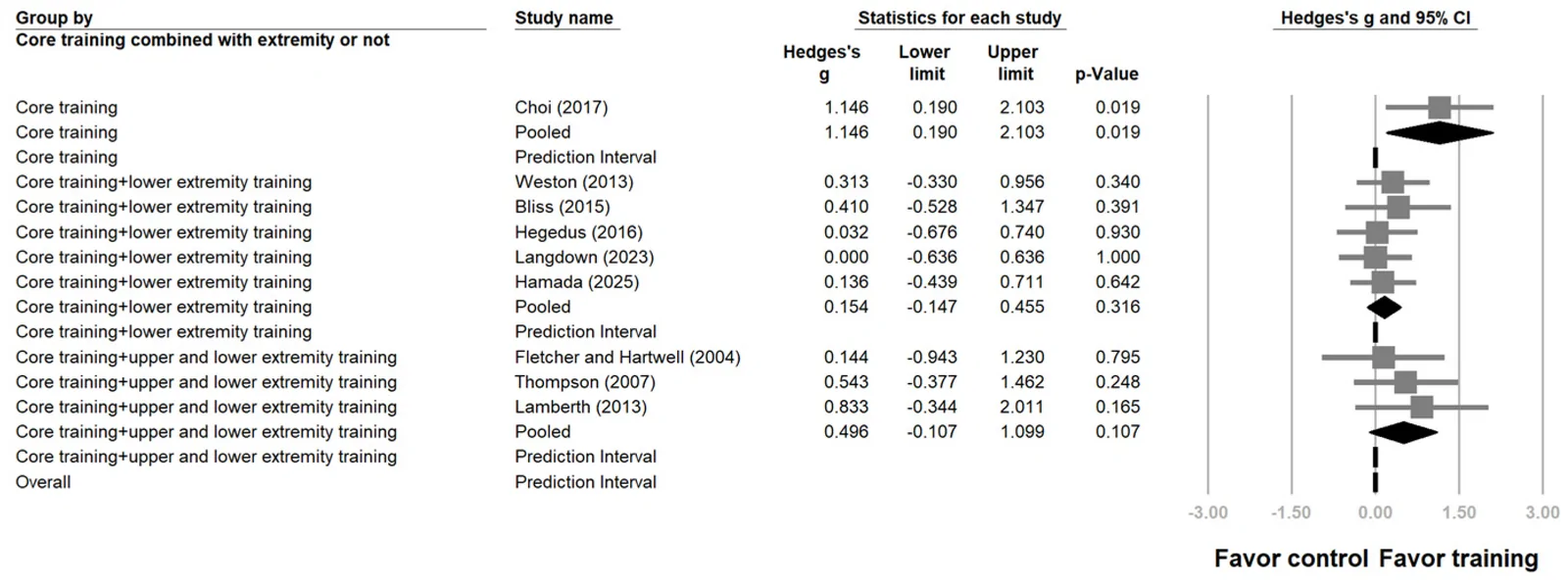
Subgroup analysis forest plot assessing the effects of core-inclusive physical training on CHS categorized by whether core training is combined with extremity training [26,28,29,31,32] or not [24,25,27].

**Figure 5:**
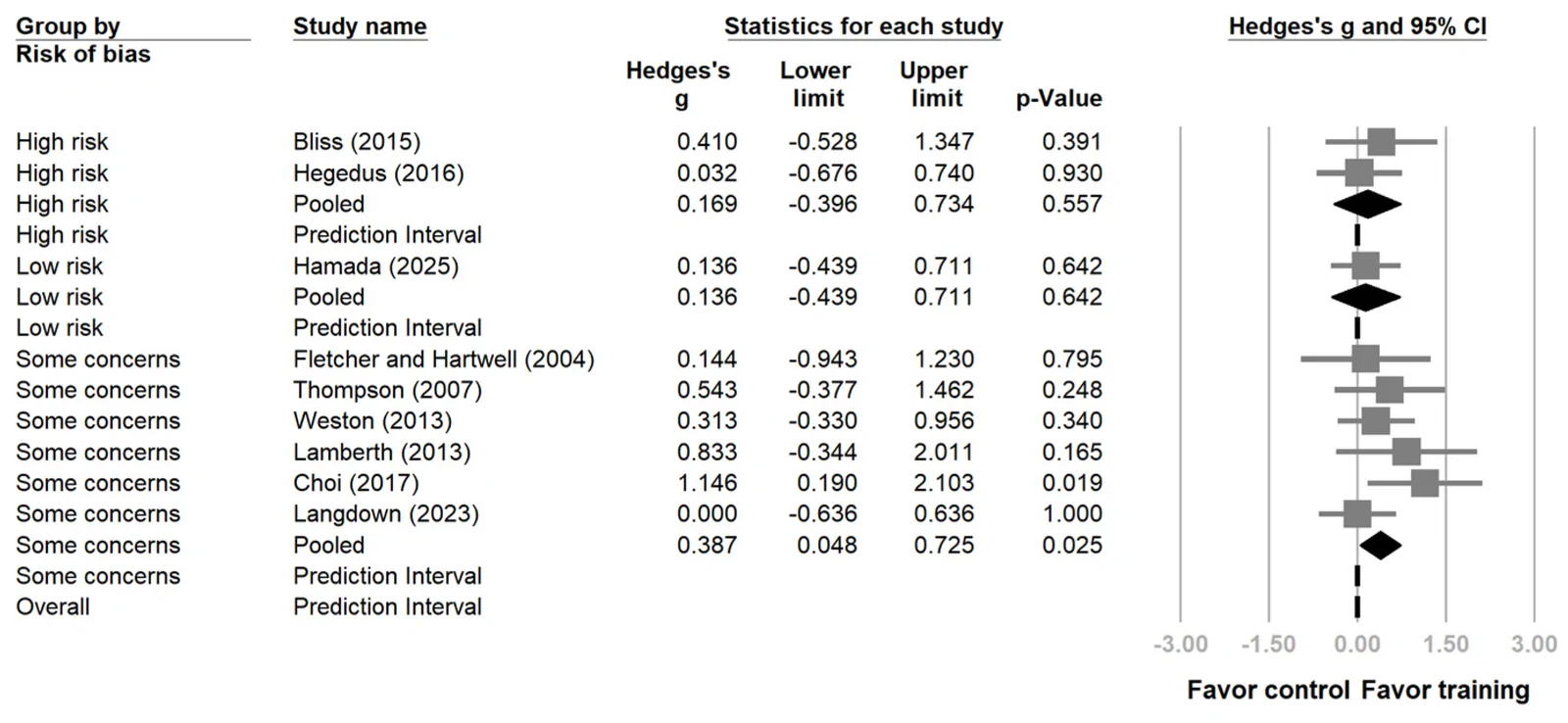
Subgroup analysis forest plot assessing the effects of core-inclusive physical training on CHS categorized by methodological quality; high risk [28,29], low risk [32] and some concerns [24,25,26,27,30,31].

**Table 1:** Characteristics of included studies in the meta-analysis.

	Country	Population	Participants (Female/Male)	Age ^1^	Study Design	Allocation Concealment	Randomization	Funding/Grants/Support
Fletcher 2004 [24]	UK	Club standard golfers	Intervention: 0/6 Control: 0/5	Whole group: 29 ± 7.4	RCT	Not mentioned	Not mentioned	Not mentioned
Thompson 2007 [25]	USA	Older male recreational golfers	Intervention: 0/11 Control: 0/7	Whole group: 70.7 ± 9.1	RCT	Not mentioned	Not mentioned	University of San Francisco’s Faculty Development Fund
Weston 2013 [26]	UK	Club-level male golfers	Intervention: 0/18 Control: 0/18	Whole group: 47 ± 12	RCT	Not mentioned	Not mentioned	None
Lamberth 2013 [27]	USA	Skilled amateur golfers (PGA golf management students)	Intervention: 0/5 Control: 0/5	Whole group: 21.4 ± 2.3	RCT	Not mentioned	Not mentioned	Not mentioned
Bliss 2015 [28]	UK	Skilled adolescent male golfers	Intervention: 0/8 Control: 0/8	Intervention: 17.3 ± 1.5 Control: 17.4 ± 0.9	Non-RCT (matched allocation according to handicap and age)	N/A ^2^	N/A ^2^	Not mentioned
Hegedus 2016 [29]	USA	Amateur female golfers	Intervention: 14/0 Control: 15/0	Intervention: 57.6 ± 3.7 Control: 58.5 ± 2.1	RCT	Sealed envelopes	Not mentioned	None
Choi 2017 [30]	Korea	Amateur golfers	Intervention: 9 (not available) Control: 9 (not available)	Intervention: 36.22 ± 5.91 Control: 35.83 ± 9.01	RCT	Not mentioned	Not mentioned	Not mentioned
Langdown 2023 [31]	UK	Mixed ability golfers	Intervention: 9/12 Control: 2/14	Intervention: 39.7 ± 11.1 Control: 39.7 ± 12.7	RCT	Not mentioned	Not mentioned	The Professional Golfers’ Association
Hamada 2025 [32]	Japan	Adolescent golfers	Intervention: 4/19 Control: 5/17	Intervention: 16.0 (16.0–17.0) Control: 16.0 (15.0–16.0)	RCT, double-blind, sham-controlled	Independent researchers	Using simple randomization with Research Randomizer	N/A ^3^

N/A, not applicable; RCT, randomized controlled trial; UK, United Kingdom; USA, United States of America. ^1^ Age is presented as means ± standard deviations or as medians (interquartile ranges). ^2^ N/A in allocation concealment and randomization indicates that this study used matched allocation rather than random assignment (i.e., non-RCT). ^3^ N/A for funding indicates that the authors explicitly stated “Not applicable” in their funding declaration.

## Data Availability

The data supporting the findings of this study are available within the article and its Supplementary Materials. Additional data are available on request from the corresponding author.

## Supplementary Materials

The following supporting information can be downloaded at: https://www.mdpi.com/article/10.3390/life16101609/s1, Table S1: PRISMA checklist; Table S2: Keywords and search results in different databases; Table S3: Details of data extraction from included randomized controlled trials; Table S4: Excluded studies and reasons; Table S5: GRADE evidence profile; Figure S1: Summary of quality assessment of studies included in the meta-analysis using Cochrane risk of bias 2 tool; Figure S2: Forest plot of sensitivity analysis excluding the non-randomized trial (RCT-only analysis); Figure S3: Forest plot of sequential leave-one-out sensitivity analysis for overall clubhead speed; Figure S4: Categorical meta-regression plot comparing intervention effects on clubhead speed between GSCT and non-GSCT subgroups; Figure S5: Forest plot of sequential leave-one-out sensitivity analysis for GSCT; Figure S6: Categorical meta-regression plot comparing intervention effects on clubhead speed across extremity conditioning integration subgroups; Figure S7: Categorical meta-regression plot evaluating intervention effects on clubhead speed across methodological quality (risk-of-bias) categories.; Figure S8: Meta-regression analysis examining the association between clubhead speed and treatment duration; Figure S9: Meta-regression analysis examining the association between clubhead speed and training frequency; Figure S10: Meta-regression analysis examining the association between clubhead speed and mean participant age; Figure S11: Meta-regression analysis examining the association between clubhead speed and proportion of male participants; Figure S12: Funnel plot of the studies evaluating clubhead speed for publication bias.

## Abbreviations

The following abbreviations are used in this manuscript:

CHS	Clubhead Speed
GSCT	Golf-specific Swing-simulated Core Training
CI	Confidence Interval
PRISMA	Preferred Reporting Items for Systematic Reviews and Meta-Analyses
SD	Standard Deviation
RoB 2	Risk of Bias 2 tool
SI	International System of Units
SE	Standard Error
MOST	Ministry of Science and Technology, Taiwan
NSTC	National Science and Technology Council, Taiwan
