# Supplementary material for: Effects of Core-Inclusive Physical Training on Clubhead Speed in Golfers: A Systematic Review and Meta-Analysis
Source: Life (Basel). 2026 Sep 25;16(10):1609. doi: 10.3390/life16101609 (PMC13706335; doi:10.3390/life16101609)
Supplement: Supplementary file 1 [file life-16-01609-s001.zip › life-4559123-supplementary.pdf]

Table S1. PRISMA Checklist

Section and Topic	#	Checklist item	Location
**TITLE**			
Title	1	Identify the report as a systematic review.	Title
**ABSTRACT**			
Abstract	2	See the PRISMA 2020 for Abstracts checklist.	Abstract
**INTRODUCTION**			
Rationale	3	Describe the rationale for the review in the context of existing knowledge.	Introduction
Objectives	4	Provide an explicit statement of the objective(s) or question(s) the review addresses.	Introduction
**METHODS**			
Eligibility criteria	5	Specify the inclusion and exclusion criteria for the review and how studies were grouped for the syntheses.	Methods
Information sources	6	Specify all databases, registers, websites, organisations, reference lists and other sources searched or consulted to identify studies. Specify the date when each source was last searched or consulted.	Methods
Search strategy	7	Present the full search strategies for all databases, registers and websites, including any filters and limits used.	Methods, Table S2
Selection process	8	Specify the methods used to decide whether a study met the inclusion criteria of the review, including how many reviewers screened each record and each report retrieved, whether they worked independently, and if applicable, details of automation tools used in the process.	Methods, Figure 1
Data collection process	9	Specify the methods used to collect data from reports, including how many reviewers collected data from each report, whether they worked independently, any processes for obtaining or confirming data from study investigators, and if applicable, details of automation tools used in the process.	Methods<br>Table S3
Data items	10a	List and define all outcomes for which data were sought. Specify whether all results that were compatible with each outcome domain in each study were sought (e.g., for all measures, time points, analyses), and if not, the methods used to decide which results to collect.	Methods, Table S3
	10b	List and define all other variables for which data were sought (e.g., participant and intervention characteristics, funding sources). Describe any assumptions made about any missing or unclear information.	Methods, Table 1-2, Table S3
Study risk of bias assessment	11	Specify the methods used to assess risk of bias in the included studies, including details of the tool(s) used, how many reviewers assessed each study and whether they worked independently, and if applicable, details of automation tools used in the process.	Methods
Effect measures	12	Specify for each outcome the effect measure(s) (e.g., risk ratio, mean difference) used in the synthesis or presentation of results.	Methods
Synthesis methods	13a	Describe the processes used to decide which studies were eligible for each synthesis (e.g., tabulating the study intervention characteristics and comparing against the planned groups for each synthesis).	Methods, Figure 1
	13b	Describe any methods required to prepare the data for presentation or synthesis, such as handling of missing summary statistics, or data conversions.	Methods, Table S3
	13c	Describe any methods used to tabulate or visually display results of individual studies and syntheses.	Methods
	13d	Describe any methods used to synthesize results and provide a rationale for the choice(s). If meta-analysis was performed, describe the model(s), method(s) to identify the presence and extent of statistical heterogeneity, and software package(s) used.	Methods
	13e	Describe any methods used to explore possible causes of heterogeneity among study results (e.g., subgroup analysis, meta-regression).	Methods
	13f	Describe any sensitivity analyses conducted to assess robustness of the synthesized results.	Methods
Reporting bias assessment	14	Describe any methods used to assess risk of bias due to missing results in a synthesis (arising from reporting biases).	Methods, Figure 11, Table 3-4

Certainty assessment	15	Describe any methods used to assess certainty (or confidence) in the body of evidence for an outcome.	Methods
		**RESULTS**	
Study selection	16a	Describe the results of the search and selection process, from the number of records identified in the search to the number of studies included in the review, ideally using a flow diagram.	Results, Figure 1,<br>Table S2, S4
	16b	Cite studies that might appear to meet the inclusion criteria, but which were excluded, and explain why they were excluded.	Results, Table S4
Study characteristics	17	Cite each included study and present its characteristics.	Results, Table 1-2
Risk of bias	18	Present assessments of risk of bias for each included study.	Figure S1, Table 3-4
Results of individual studies	19	For all outcomes, present, for each study: (a) summary statistics for each group (where appropriate) and (b) an effect estimates and its precision (e.g., confidence/credible interval), ideally using structured tables or plots.	Figure 2-5, Figure S2-S5
Results of syntheses	20a	For each synthesis, briefly summarise the characteristics and risk of bias among contributing studies.	Results, Table 3-4
	20b	Present results of all statistical syntheses conducted. If meta-analysis was done, present for each the summary estimate and its precision (e.g., confidence/credible interval) and measures of statistical heterogeneity. If comparing groups, describe the direction of the effect.	Figure 2-5, Figure S2-5
	20c	Present results of all investigations of possible causes of heterogeneity among study results.	Figure 3-5, Figure S6-12
	20d	Present results of all sensitivity analyses conducted to assess the robustness of the synthesized results.	Results, Figure S2-5
Reporting biases	21	Present assessments of risk of bias due to missing results (arising from reporting biases) for each synthesis assessed.	Figure S1, Table 3-4
Certainty of evidence	22	Present assessments of certainty (or confidence) in the body of evidence for each outcome assessed.	Table S5
		**DISCUSSION**	
Discussion	23a	Provide a general interpretation of the results in the context of other evidence.	Discussion
	23b	Discuss any limitations of the evidence included in the review.	Discussion
	23c	Discuss any limitations of the review processes used.	Discussion
	23d	Discuss implications of the results for practice, policy, and future research.	Discussion
		**OTHER INFORMATION**	
Registration and protocol	24a	Provide registration information for the review, including register name and registration number, or state that the review was not registered.	Methods
	24b	Indicate where the review protocol can be accessed, or state that a protocol was not prepared.	Method
	24c	Describe and explain any amendments to information provided at registration or in the protocol.	Methods
Support	25	Describe sources of financial or non-financial support for the review, and the role of the funders or sponsors in the review.	Funding
Competing interests	26	Declare any competing interests of review authors.	Conflicts of Interest
Availability of data, code and other materials	27	Report which of the following are publicly available and where they can be found: template data collection forms; data extracted from included studies; data used for all analyses; analytic code; any other materials used in the review.	Results, Table S1-S5

Table S2. Keywords and search results in different databases

Database	Keyword	Filter	Date	Results
PubMed	("Golf" OR "Golfer" OR "Golfers" OR "Golf swing") AND ("Core training" OR "Core stability" OR "Core exercise" OR "Trunk training" OR "Trunk stability" OR "Lumbopelvic" OR "Spinal stabilization" OR "Plyometric" OR "Resistance training" OR "Strength training" OR "Functional training" OR "Corrective exercise" OR "Weight ball" OR "Medicine ball")	N/A	To 30 June 2026	113
Embase	("Golf" OR "Golfer" OR "Golfers" OR "Golf swing") AND ("Core training" OR "Core stability" OR "Core exercise" OR "Trunk training" OR "Trunk stability" OR "Lumbopelvic" OR "Spinal stabilization" OR "Plyometric" OR "Resistance training" OR "Strength training" OR "Functional training" OR "Corrective exercise" OR "Weight ball" OR "Medicine ball")	N/A	To 30 June 2026	227
Cochrane CENTRAL	("Golf" OR "Golfer" OR "Golfers" OR "Golf swing") AND ("Core training" OR "Core stability" OR "Core exercise" OR "Trunk training" OR "Trunk stability" OR "Lumbopelvic" OR "Spinal stabilization" OR "Plyometric" OR "Resistance training" OR "Strength training" OR "Functional training" OR "Corrective exercise" OR "Weight ball" OR "Medicine ball")	N/A	To 30 June 2026	31
Web of Science	("Golf" OR "Golfer" OR "Golfers" OR "Golf swing") AND ("Core training" OR "Core stability" OR "Core exercise" OR "Trunk training" OR "Trunk stability" OR "Lumbopelvic" OR "Spinal stabilization" OR "Plyometric" OR "Resistance training" OR "Strength training" OR "Functional training" OR "Corrective exercise" OR "Weight ball" OR "Medicine ball")	N/A	To 30 June 2026	141
Scopus	("Golf" OR "Golfer" OR "Golfers" OR "Golf swing") AND ("Core training" OR "Core stability" OR "Core exercise" OR "Trunk training" OR "Trunk stability" OR "Lumbopelvic" OR "Spinal stabilization" OR "Plyometric" OR "Resistance training" OR "Strength training" OR "Functional training" OR "Corrective exercise" OR "Weight ball" OR "Medicine ball")	N/A	To 30 June 2026	107
PEDro	Golf (Simple Search)	N/A	To 30 June 2026	21
Googlesholar	("Golf" OR "Golfer" OR "Golfers" OR "Golf swing") AND ("Core training" OR "Core stability" OR "Core exercise" OR "Trunk training" OR "Trunk stability" OR "Lumbopelvic" OR "Spinal stabilization" OR "Plyometric" OR "Resistance training" OR "Strength training" OR "Functional training" OR "Corrective exercise" OR "Weight ball" OR "Medicine ball")	First 200 records were screened	To 30 June 2026	200

N/A: not applied

Table S3. Details of data extraction from included randomized controlled trials

First Author & Year	Data Extraction Details	Sample Size & Participant Characteristics	Outcome Measurements (Pre vs. Post Means ± SDs)	Conversion & Methodological Rationale
Fletcher 2004	Extraction: Data extracted from Table 2. Sample size derived from 'Subjects' section.<br>Club Type: Driver (Maximal velocity)	Total N = 11<br>(INT n = 6, CON n = 5)	INT Pre: 179.8 ± 9.1 km/h<br>INT Post: 182.6 ± 6.2 km/h<br>CON Pre: 172.3 ± 17.1 km/h<br>CON Post: 173.0 ± 18.7 km/h	Reported in km/h. Converted to m/s for SI unit standardization. Change score SD derived assuming r = 0.5.
Thompson 2007	Extraction: Data extracted from Abstract (consistent with Figure 5 visual data). Results section text error for post-test INT SD (21.6) excluded to maintain integrity.<br>Club Type: Driver	Total N = 18<br>(INT n = 11, CON n = 7)	INT Pre: 127.3 ± 13.4 km/h<br>INT Post: 133.6 ± 14.2 km/h<br>CON Pre: 134.5 ± 14.6 km/h<br>CON Post: 133.3 ± 11.2 km/h	Reported in km/h. Converted to m/s. Post-intervention INT SD was verified against Figure 5 error bars. Change score SD derived assuming r = 0.5.
Weston 2013	Extraction: Baseline CHS and sample size extracted from Abstract/Methods. Table 2 provided ANCOVA-adjusted % change (+2.0% INT, -1.5% CON).<br>Club Type: 5-iron	Total N = 36<br>(INT n = 18, CON n = 18)	INT Pre: 79.9 ± 8.4 mph<br>Calculated Post: 81.5 ± 8.4 mph<br>CON Pre: 77.6 ± 8.8 mph<br>Calculated Post: 76.4 ± 8.8 mph	Reported in mph. Converted to m/s. Post-intervention means calculated from ANCOVA % change. Post-test SDs substituted with baseline SDs per Cochrane guidelines as CIs represented difference in change scores.
Lamberth 2013	Extraction: CHS data extracted directly from Results section text and Figure 1.<br>Club Type: Driver	Total N = 10<br>(INT n = 5, CON n = 5)	INT Pre: 50.87 ± 3.62 m/s<br>INT Post: 48.91 ± 1.48 m/s<br>CON Pre: 47.21 ± 1.56 m/s<br>CON Post: 46.31 ± 0.67 m/s	Natively reported in m/s. Change score SD derived assuming r = 0.5.
Bliss 2015	Extraction: CHS data extracted from Table 2. Sample size derived from Participants section.<br>Club Type: 5-iron	Total N = 16<br>(INT n = 8, CON n = 8)	INT Pre: 133.9 ± 11.1 km/h<br>INT Post: 137.9 ± 10.5 km/h<br>CON Pre: 136.2 ± 12.2 km/h<br>CON Post: 135.2 ± 12.5 km/h	Reported in km/h. Converted to m/s. Standard data filtering removed 31 extreme mishits (~9.7% of 320 swings) without affecting subject-level sample completeness.
Hegedus 2016	Extraction: CHS data extracted from Table 2. Reported SE converted to SD using SD = SE * sqrt(N). Driver data prioritized over 7-iron for outcome hierarchy.<br>Club Type: Driver	Total N = 29<br>(INT/GSRT n = 14, CON/TRAD n = 15)	GSRT Pre: 30.2 ± 0.8 m/s (SE)<br>GSRT Post: 30.8 ± 0.8 m/s (SE)<br>TRAD Pre: 28.3 ± 0.8 m/s (SE)<br>TRAD Post: 29.0 ± 0.8 m/s (SE)	Natively reported in m/s. SE converted to SD using Cochrane formula SD = SE * sqrt(N). Driver CHS was extracted per pre-specified outcome hierarchy to evaluate maximal velocity.
Choi 2017	Extraction: CHS data extracted directly from Table 2.<br>Club Type: 7-iron	Total N = 18<br>(INT n = 9, CON n = 9)	INT Pre: 37.66 ± 3.74 m/s<br>INT Post: 43.66 ± 4.03 m/s<br>CON Pre: 38.66 ± 5.40 m/s<br>CON Post: 39.22 ± 4.96 m/s	Natively reported in m/s. Raw pre- and post-intervention descriptive statistics directly extracted. Isolated core rotational throw protocol.
Langdown 2023	Extraction: CHS data extracted from Table 5.<br>Club Type: 6-iron	Total N = 37<br>(INT n = 21, CON n = 16)	INT Pre: 33.0 ± 6.5 m/s<br>INT Post: 32.7 ± 6.0 m/s<br>CON Pre: 36.2 ± 4.3 m/s<br>CON Post: 35.9 ± 4.1 m/s	Natively reported in m/s. 6-iron TrackMan launch monitor metrics extracted as driver data were not collected.

Hamada 2025	Extraction: CHS data extracted from Table 3.<br>Club Type: Driver	Total N = 45<br>(INT/GLEP n = 23, CON/Sham n = 22)<br>Adolescent golfers	GLEP Pre: 44.0 ± 5.6 m/s<br>GLEP Post (W12): 42.8 ± 5.6 m/s<br>Sham Pre: 43.0 ± 4.4 m/s<br>Sham Post (W12): 42.5 ± 4.4 m/s	Natively reported in m/s. Final Week 12 post-intervention evaluation extracted. Missing values handled via linear mixed-model (LMM) ANOVA without excluding subjects.

Abbreviations: ANCOVA = Analysis of covariance; CHS = Clubhead speed; CON = Control group; GLEP = Golfer's low back pain exercise prevention program; GSRT = Golf-specific resistance training; INT = Intervention group; km/h = Kilometers per hour; m/s = Meters per second; mph = Miles per hour; N = Total sample size; n = Subgroup sample size; SD = Standard deviation; SE = Standard error; SI = International System of Units; TRAD = Traditional resistance training.

1. Unit Standardization (m/s): All velocity measurements were standardized to meters per second (m/s) using exact conversion factors (1 km/h = 0.27778 m/s; 1 mph = 0.44704 m/s).

2. Effect Size Computation (Hedges' g & Change Scores): Effect sizes were calculated using pre-to-post change scores between intervention and control groups. Standard deviations of change scores (SD_change) were derived using the formula**: $SD\_\Delta = \sqrt{(SD\_pre^2 + SD\_post^2 - 2 \cdot r \cdot SD\_pre \cdot SD\_post)}$,** with an assumed pre-post correlation coefficient (r) of 0.5 in accordance with Cochrane Handbook guidelines. Standardized mean difference (Hedges' g) with small-sample bias correction was preferred over raw mean difference to standardize treatment effects relative to study-specific variability across different club types.

3. Outcome Selection Hierarchy for Club Types: To address methodological heterogeneity arising from different golf clubs, data from the driver were prioritized to evaluate maximal swing velocity (Fletcher 2004, Thompson 2007, Lamberth 2013, Hegedus 2016, Hamada 2025). When driver metrics were unavailable, long or mid-irons were selected hierarchically (5-iron in Weston 2013 and Bliss 2015; 6-iron in Langdown 2023; 7-iron in Choi 2017).

4. Meta-Analytic Model & Variance Estimator: DerSimonian and Laird random-effects model was employed for effect size pooling. Between-study variance was estimated as $tau^2 = 0.000$ (Cochran Q = 5.049, df = 7, p = 0.654, $I^2 = 0.0\%$), confirming low statistical heterogeneity across included trials.

Table S4. Excluded studies and reasons

Citations	Reasons
Thompson, C. J., & Osness, W. H. (2004). Effects of an 8-Week Multimodal Exercise Program on Strength, Flexibility, and Golf Performance in 55- to 79-Year-Old Men. *Journal of Aging and Physical Activity*, 11, 144-156.	Although the study examined the effects of an exercise intervention on golf clubhead speed (CHS), it lacks a dedicated data table for this primary outcome measure. Furthermore, the text reports only the mean CHS values for both groups without providing the corresponding standard deviations (SD) or exact ANCOVA F-values. Consequently, the lack of crucial variance data makes it mathematically impossible to calculate the poolable effect sizes required for our quantitative synthesis.
Coughlan, D., Taylor, M. J. D., Wayland, W., Brooks, D., & Jackson, J. (2020). The effect of a 12-week strength and conditioning programme on youth golf performance. *International Journal of Golf Science*, 8(1).	The experimental design incorporated elements of cross-over and repeated measures, subjecting participants to both intervention and control conditions. This introduces significant risks of carryover and learning effects, conflicting with the methodological requirement for an independent parallel-group design.
Oranchuk, D. J., Mannerberg, J. M., Robinson, T. L., & Nelson, M. C. (2018). Eight weeks of strength and power training improves club head speed in collegiate golfers. *The Journal of Strength and Conditioning Research*, *34*(8), 2205–2213.	The training protocols of both the experimental and control cohorts actively recruited and conditioned the core musculature through either high-load isometric stabilization or dynamic rotatio[1]nal exercises. This introduces significant risks of treatment contamination, conflicting with the methodological requirement to isolate the independent, net therapeutic impact of core muscle training against a non-exposed control condition.
Li, M., Ruan, C., & Zhang, L. (2024). Impact of Four Weeks of TOGU Training on Neuromuscular Control and Golf Swing Performance. *Journal of Functional Morphology and Kinesiology*, 9(4), 243.	The relevant golf performance outcomes in this study were exclusively presented as visual graphs. In the absence of precise numerical data tables (means ± SDs), accurate extraction of continuous variables for statistical conversion and quantitative synthesis was not feasible.
Shaw, J., Gould, Z. I., Oliver, J. L., & Lloyd, R. S. (2024). Twelve Weeks of Progressive Resistance Training Positively Improves Physical Fitness and Golf Swing Performance in Talented Youth Golfers. *Journal of Strength and Conditioning Research*.	Data pertaining to clubhead speed were solely presented using line graphs and effect sizes. The study did not provide comprehensive pre- and post-intervention descriptive statistics (means ± SDs), thus lacking the exact numerical data requisite for meta-analysis.
Hamada, Y., Akasaka, K., Otsudo, T., Sawada, Y., Hattori, H., Kikuchi, Y., & Hall, T. (2025). Golfers' Performance Is Improved More by Combining Foam Rolling and Dynamic Stretch to the Lead Hip Than Practice Golf Swinging. *Journal of Strength and Conditioning Research*.	This study employed a randomized crossover trial design. Participants received a combined foam rolling and dynamic stretching intervention and subsequently crossed over to conventional golf swing practice. This design cannot provide data from independent parallel comparison groups, which violates the predefined inclusion criteria for parallel-group trials..

Table S5 GRADE evidence profile

A. Evidence Characteristics and Pooled Treatment Effects

Outcome & Comparison	Studies / Participants	Study Design	Standardized Effect Hedges' g (95% CI)
Overall Core-Inclusive Physical Training	9 trials; 220 participants	8 randomized controlled trials (RCTs); 1 non-randomized trial (Bliss 2015)	Hedges' g: 0.290 (0.031 to 0.549) [p = 0.028, I² = 0.0%]
Golf-Specific Swing-Simulated Core Training (GSCT) vs. Control	5 trials; 114 participants	4 randomized controlled trials; 1 non-randomized trial (Bliss 2015)	Hedges' g: 0.451 (0.073 to 0.830) [p = 0.020, I² = 0.0%]
Non-GSCT Core Regimens vs. Control	4 trials; 106 participants	4 randomized controlled trials (Weston, Lamberth, Langdown, Hamada)	Hedges' g: 0.148 (-0.208 to 0.503) [p = 0.415, I² = 0.0%]

B. Detailed GRADE Certainty-Domain Judgments

Outcome & Comparison	Starting Certainty	Risk of Bias (Domain 1)	Inconsistency (Domain 2)	Indirectness (Domain 3)	Imprecision (Domain 4)	Publication Bias (Domain 5)	Final Rating
Overall Core-Inclusive Physical Training vs. Control (k = 9, N = 220)	High ⊕⊕⊕⊕ (8/9 RCTs)	Serious (-1) [b]	Not Serious (0) [e]	Not Serious (0) [f]	Serious (-1) [g]	No Downgrade (0) [h]	Low [a,b] ⊕⊕◯◯
Golf-Specific Swing-Simulated Core Training (GSCT) (k = 5, N = 114)	High ⊕⊕⊕⊕ (4/5 RCTs)	Serious (-1) [b]	Not Serious (0) [e]	Not Serious (0) [f]	Very Serious (-2) [i]	No Downgrade (0) [h]	Very Low [a,c] ⊕◯◯◯
Non-GSCT Core Regimens vs. Control (k = 4, N = 106)	High ⊕⊕⊕⊕ (4/4 RCTs)	Serious (-1) [b]	Not Serious (0) [e]	Not Serious (0) [f]	Serious (-1) [j]	No Downgrade (0) [h]	Low [a,d] ⊕⊕◯◯

a. Starting Certainty Assessment: Both the overall evidence body (k = 9) and subgroup bodies of evidence were initialized at High certainty because randomized controlled trials (RCTs) contributed the overwhelming majority of participants (>92.7% of total sample) and meta-analytic weight (RCT weight = 92.4% in primary random-effects model). Methodological limitations arising from the inclusion of one non-randomized study (Bliss et al., 2015) were evaluated under Domain 1 (Risk of Bias) and verified via an RCT-only sensitivity analysis (k = 8, Hedges' g = 0.280, 95% CI: 0.010 to 0.550, p = 0.042).

b. Risk of Bias (Downgraded -1 level to Serious): Seven of the eight included RCTs were rated as having 'some concerns' on the Cochrane RoB 2 tool due to incomplete reporting of allocation sequence concealment, absence of prospective clinical trial registration/Statistical Analysis Plan (SAP) prior to unblinded analysis, and unblinded participant delivery inherent to physical exercise trials.

c. GSCT Imprecision & Fragility (Downgraded -2 levels to Very Serious): Although GSCT yielded a statistically significant within-subgroup effect (k = 5, Hedges' g = 0.451, 95% CI: 0.073 to 0.830, p = 0.020), certainty was downgraded by two levels due to extreme inferential fragility and small sample size (N = 114). Specifically: (1) the pooled effect was heavily driven by a single highly influential trial (Choi et al., 2017; g = 1.146); removing Choi et al. in a subgroup-specific leave-one-out sensitivity analysis rendered the GSCT effect non-significant (k = 4, Hedges' g = 0.322, 95% CI: -0.090 to 0.734, p = 0.126); (2) formal testing for subgroup interaction against Non-GSCT was statistically non-significant (Qb = 1.31, df = 1, p = 0.252), confirming that superiority over Non-GSCT cannot be formally established; and (3) total sample size fell substantially below optimal information size (OIS) thresholds.

d. Non-GSCT Imprecision (Downgraded -1 level to Serious): Downgraded by one level because the 95% confidence interval crossed the null effect line (Hedges' g = 0.148, 95% CI: -0.208 to 0.503, p = 0.415), denoting substantial statistical imprecision surrounding the point estimate across four small RCTs (N = 106).

e. Inconsistency (No Downgrade): Between-study statistical heterogeneity was exceptionally low across all primary and subgroup models ($I^2 = 0.000\%$, $tau^2 = 0.000$; Cochran's Q = 5.910, df = 8, p = 0.657 for overall model; Q = 1.83, p = 0.767 for GSCT). Point estimates consistently aligned in a positive direction, indicating consistent treatment trajectories across varied study settings.

f. Indirectness (No Downgrade): Included trials directly addressed the target PICO question without material population, intervention, or outcome indirectness. Participants were healthy golfers across recreational, collegiate, and competitive skill tiers; interventions evaluated structured core conditioning; controls comprised active golf practice or sham regimens; and CHS was quantitatively recorded in m/s using calibrated launch monitors or 3D optical kinematics.

g. Overall Imprecision (Downgraded -1 level to Serious): Downgraded by one level because: (1) total aggregate sample size (N = 220 across 9 trials) remained below standard optimal information size recommendations (~400 participants for continuous sports performance metrics); (2) the lower bound of the 95% confidence interval approached the null threshold (lower CI = 0.031); and (3) sequential leave-one-out sensitivity analysis revealed inferential fragility, wherein omitting Choi et al. (2017) shifted the overall pooled effect from statistical significance (p = 0.028) to non-significance (k = 8, Hedges' g = 0.222, 95% CI: -0.047 to 0.491, p = 0.106).

h. Publication Bias (No Downgrade): Funnel plot inspection revealed mild visual asymmetry, and Egger's linear regression test yielded a borderline result (p = 0.057). However, in accordance with Cochrane Handbook guidance (Chapter 13 & 14), formal testing for funnel plot asymmetry is severely underpowered and unreliable when synthesis involves fewer than 10 studies (k = 9 for overall; k = 5 for GSCT). Publication bias could not be definitively ruled out, but available quantitative metrics did not justify formal downgrading.

Supplementary Figures

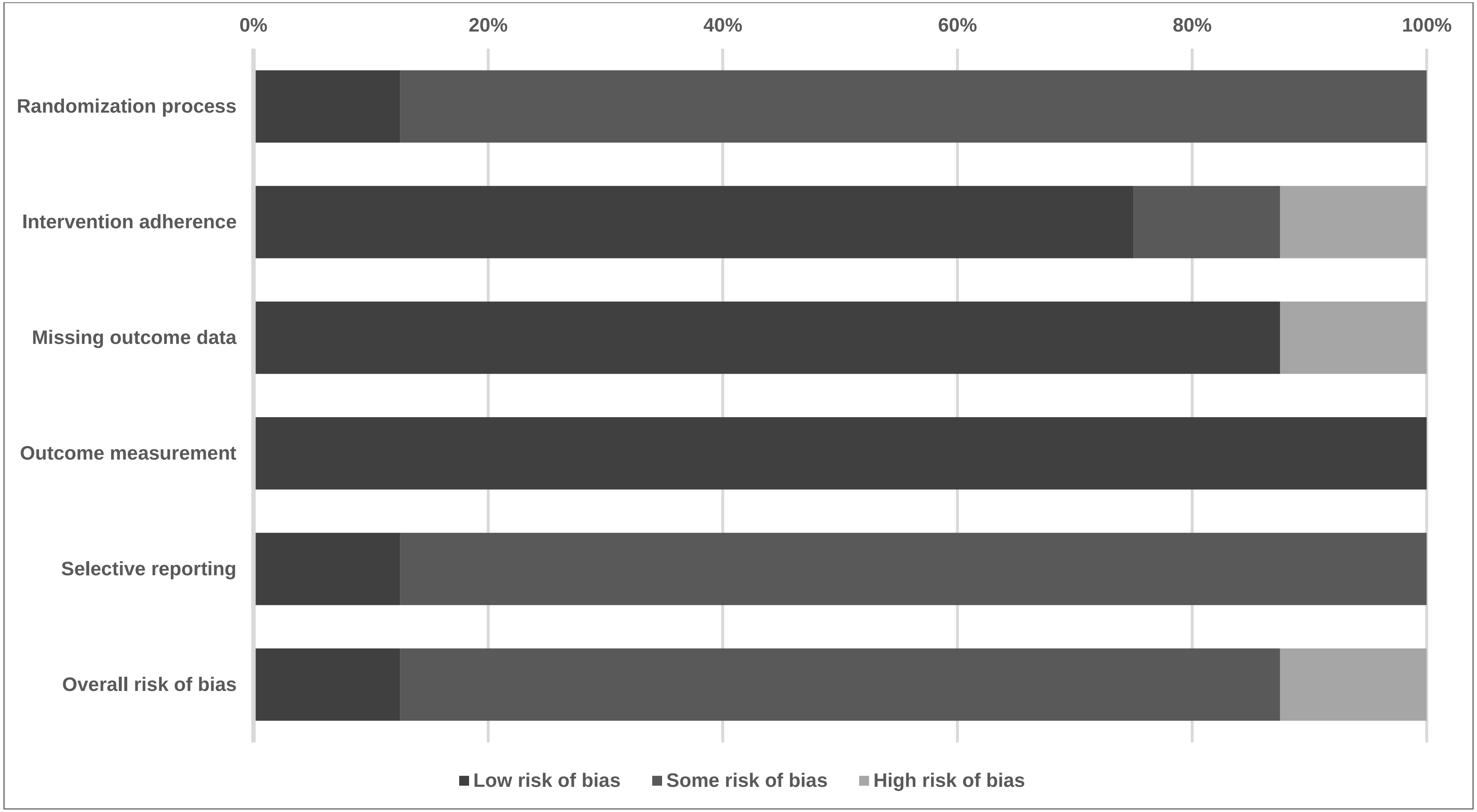

Figure S1. Summary of quality assessment of studies included in the meta-analysis using Cochrane risk of bias 2 tool

Study name	Hedges's g	Lower limit	Upper limit	p-Value
Fletcher and Hartwell (2004)	0.144	-0.943	1.230	0.795
Thompson (2007)	0.543	-0.377	1.462	0.248
Weston (2013)	0.313	-0.330	0.956	0.340
Lamberth (2013)	0.833	-0.344	2.011	0.165
Hegedus (2016)	0.032	-0.676	0.740	0.930
Choi (2017)	1.146	0.190	2.103	0.019
Langdown (2023)	0.000	-0.636	0.636	1.000
Hamada (2025)	0.136	-0.439	0.711	0.642
Pooled	0.280	0.010	0.550	0.042
Prediction Interval				

(Statistics for each study)

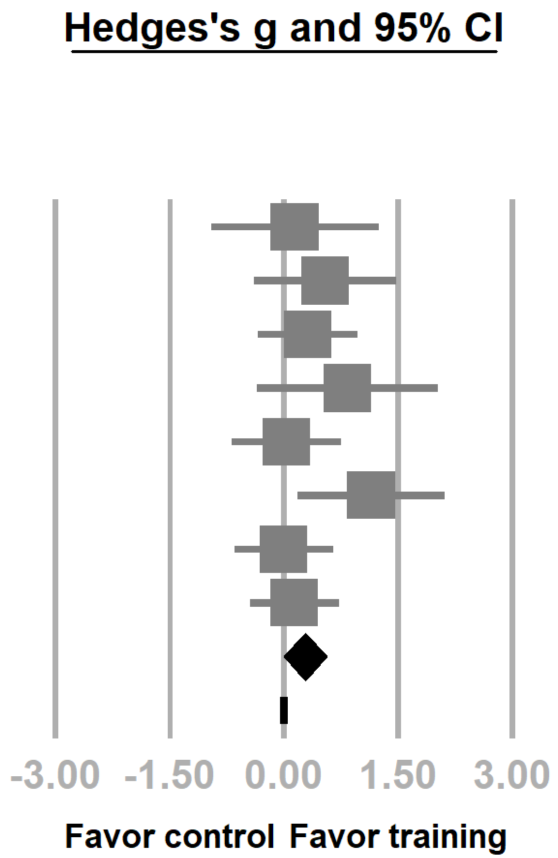

Figure S2. Forest plot of sensitivity analysis excluding the non-randomized trial (RCT-only analysis)

Study name	Statistics with study removed			
	Point	Lower limit	Upper limit	p-Value
Fletcher and Hartwell (2004)	0.299	0.032	0.566	0.028
Thompson (2007)	0.268	-0.002	0.538	0.052
Weston (2013)	0.285	0.002	0.569	0.048
Lamberth (2013)	0.262	-0.003	0.528	0.053
Bliss (2015)	0.280	0.010	0.550	0.042
Hegedus (2016)	0.330	0.051	0.608	0.020
Choi (2017)	0.222	-0.047	0.491	0.106
Langdown (2023)	0.348	0.064	0.631	0.016
Hamada (2025)	0.329	0.039	0.619	0.026
Pooled	0.290	0.031	0.549	0.028
Prediction Interval	0.000	0.000	0.000	0.000

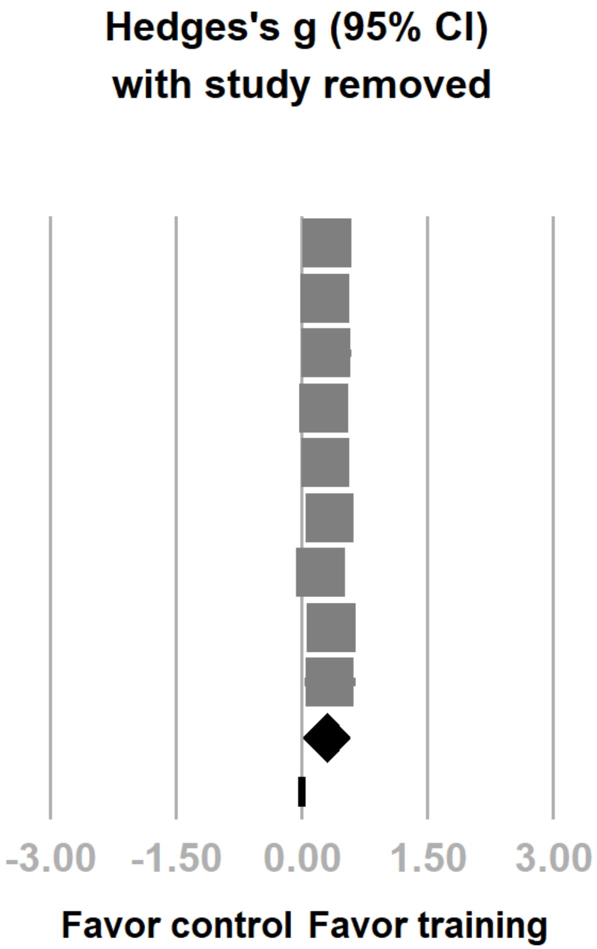

Figure S3. Forest plot of sequential leave-one-out sensitivity analysis for overall clubhead speed

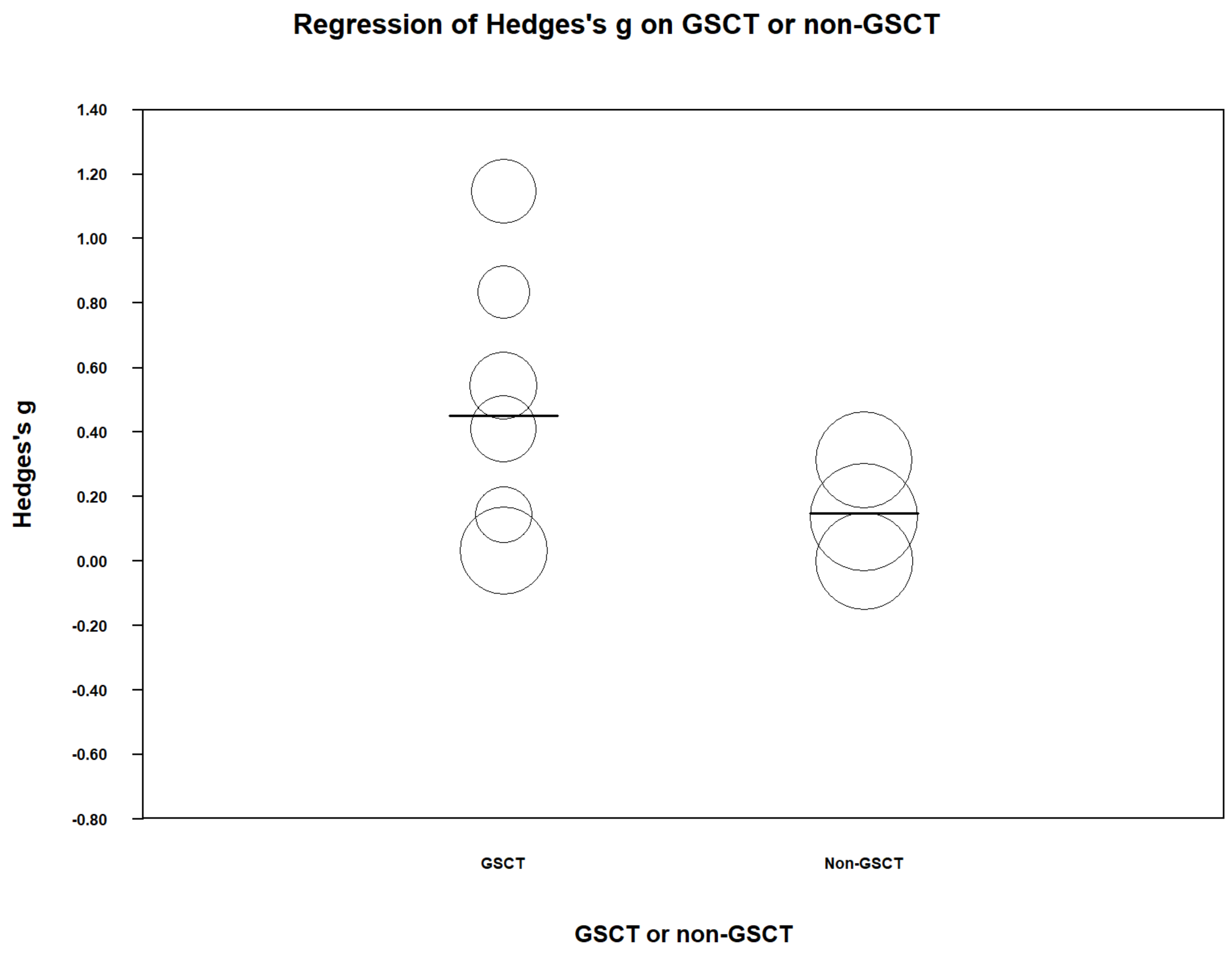

Figure S4. Categorical meta-regression plot comparing intervention effects on clubhead speed between GSCT and non-GSCT subgroups

Study name	Statistics with study removed			
	Point	Lower limit	Upper limit	p-Value
Fletcher and Hartwell (2004)	0.494	0.090	0.898	0.017
Thompson (2007)	0.435	0.014	0.856	0.043
Lamberth (2013)	0.407	0.007	0.807	0.046
Bliss (2015)	0.463	0.041	0.884	0.031
Hegedus (2016)	0.619	0.171	1.067	0.007
Choi (2017)	0.322	-0.090	0.734	0.126
Pooled	0.451	0.073	0.830	0.020
Prediction Interval	0.000	0.000	0.000	0.000

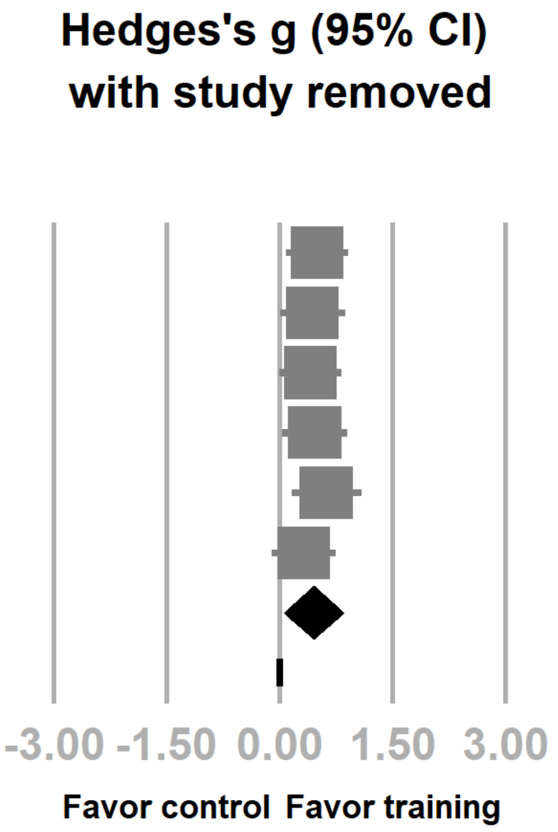

Figure S5. Forest plot of sequential leave-one-out sensitivity analysis for GSCT

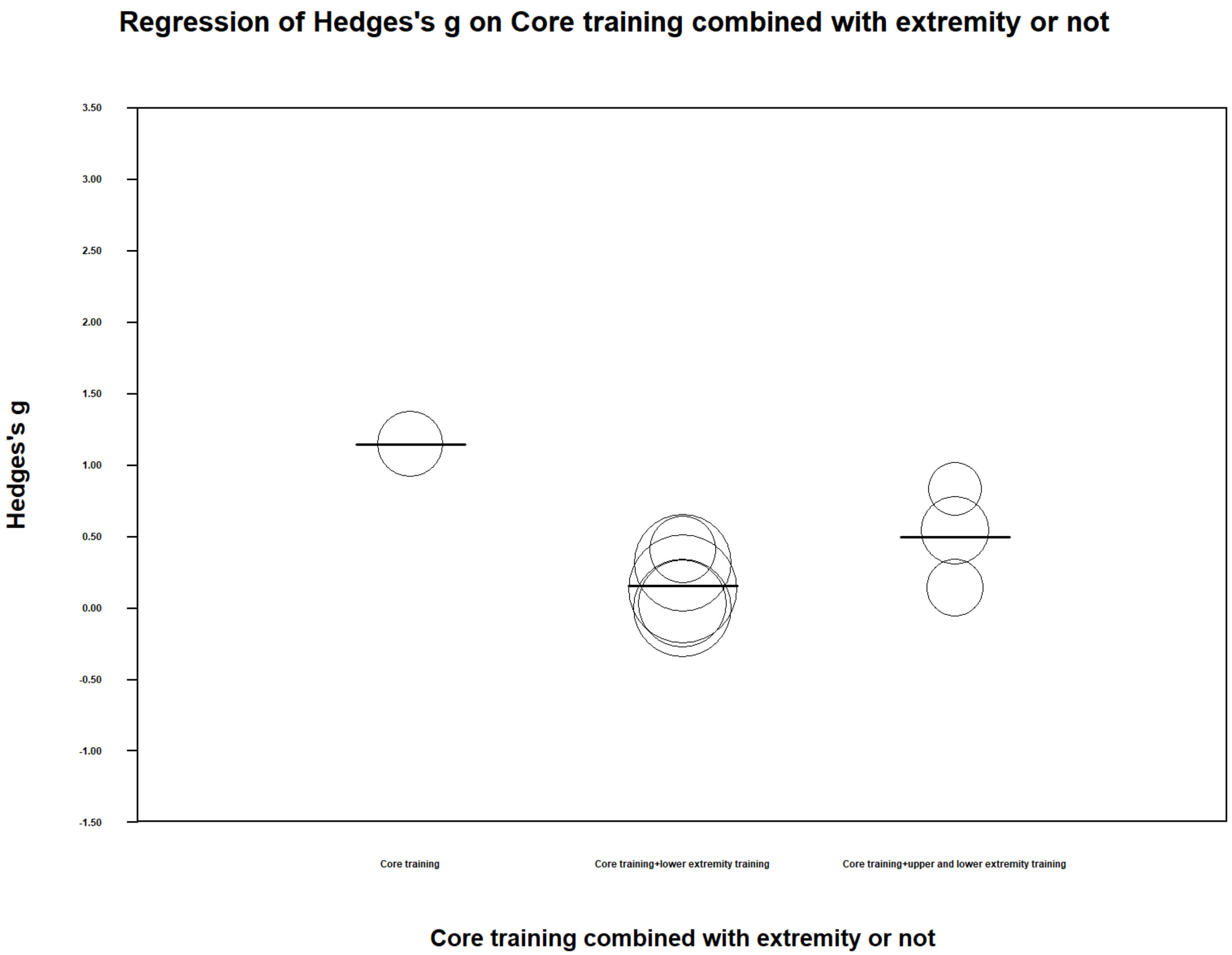

Figure S6. Categorical meta-regression plot comparing intervention effects on clubhead speed across extremity conditioning integration subgroups

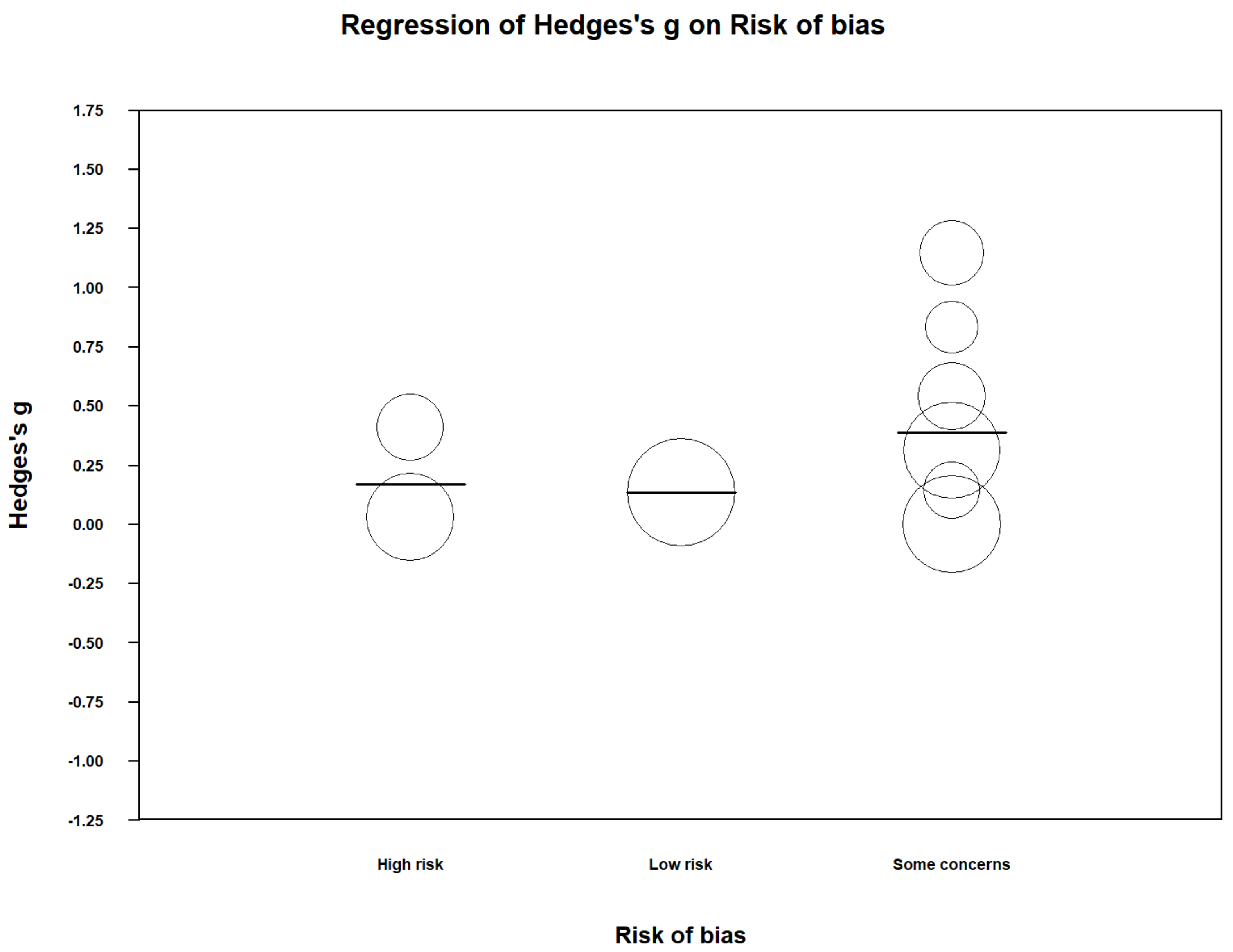

Figure S7. Categorical meta-regression plot evaluating intervention effects on clubhead speed across methodological quality (risk-of-bias) categories.

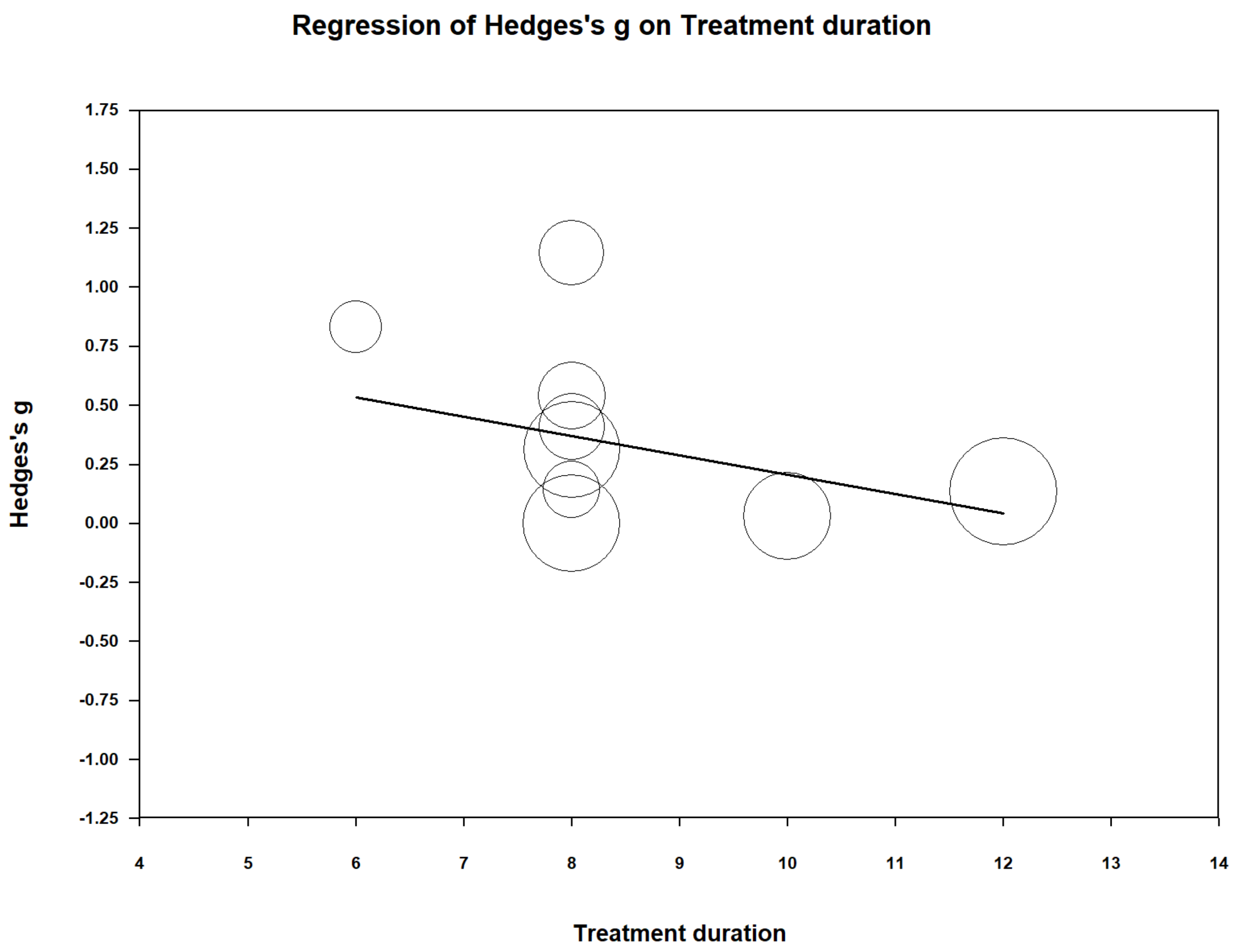

Figure S8. Meta-regression analysis examining the association between clubhead speed and treatment duration

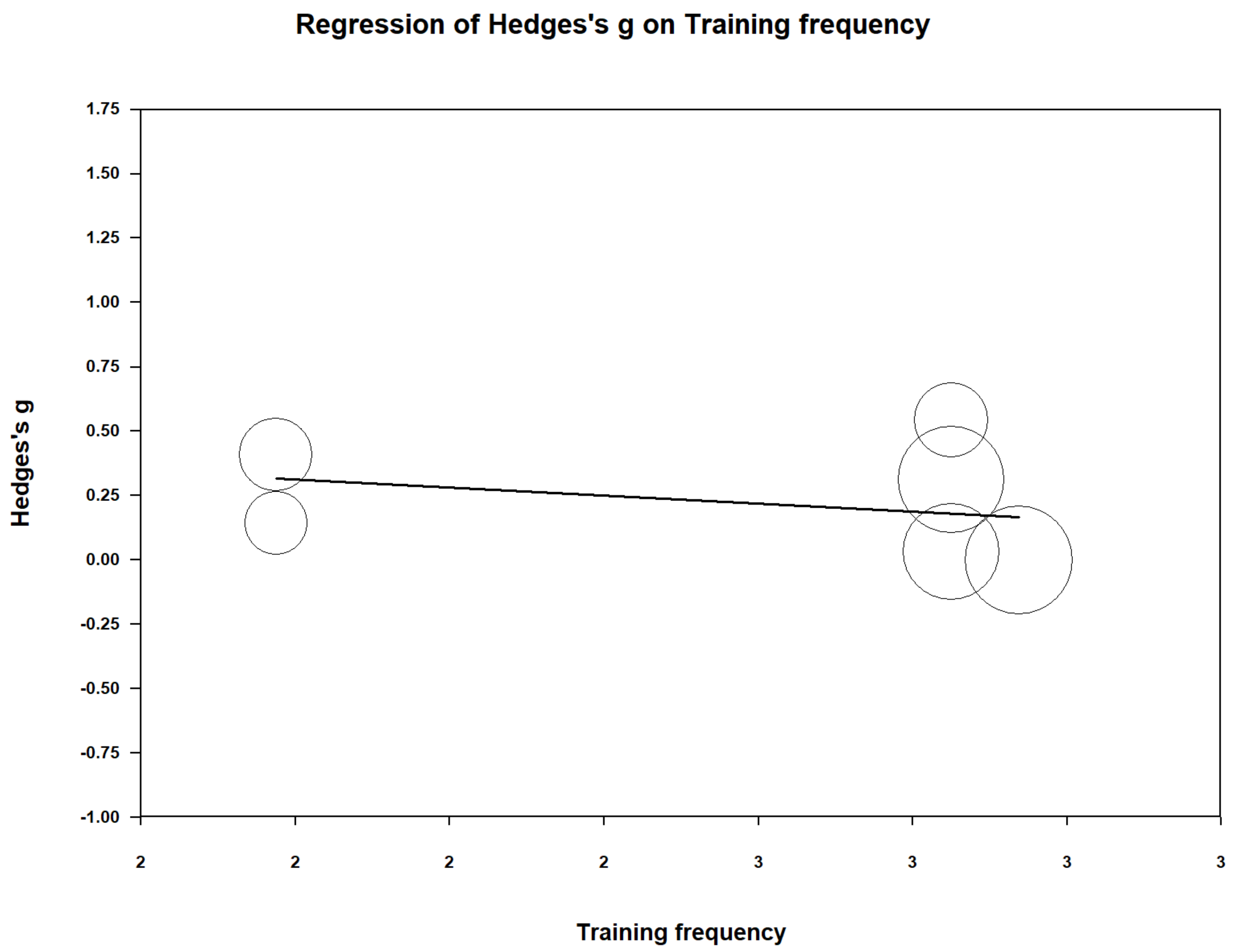

Figure S9. Meta-regression analysis examining the association between clubhead speed and training frequency

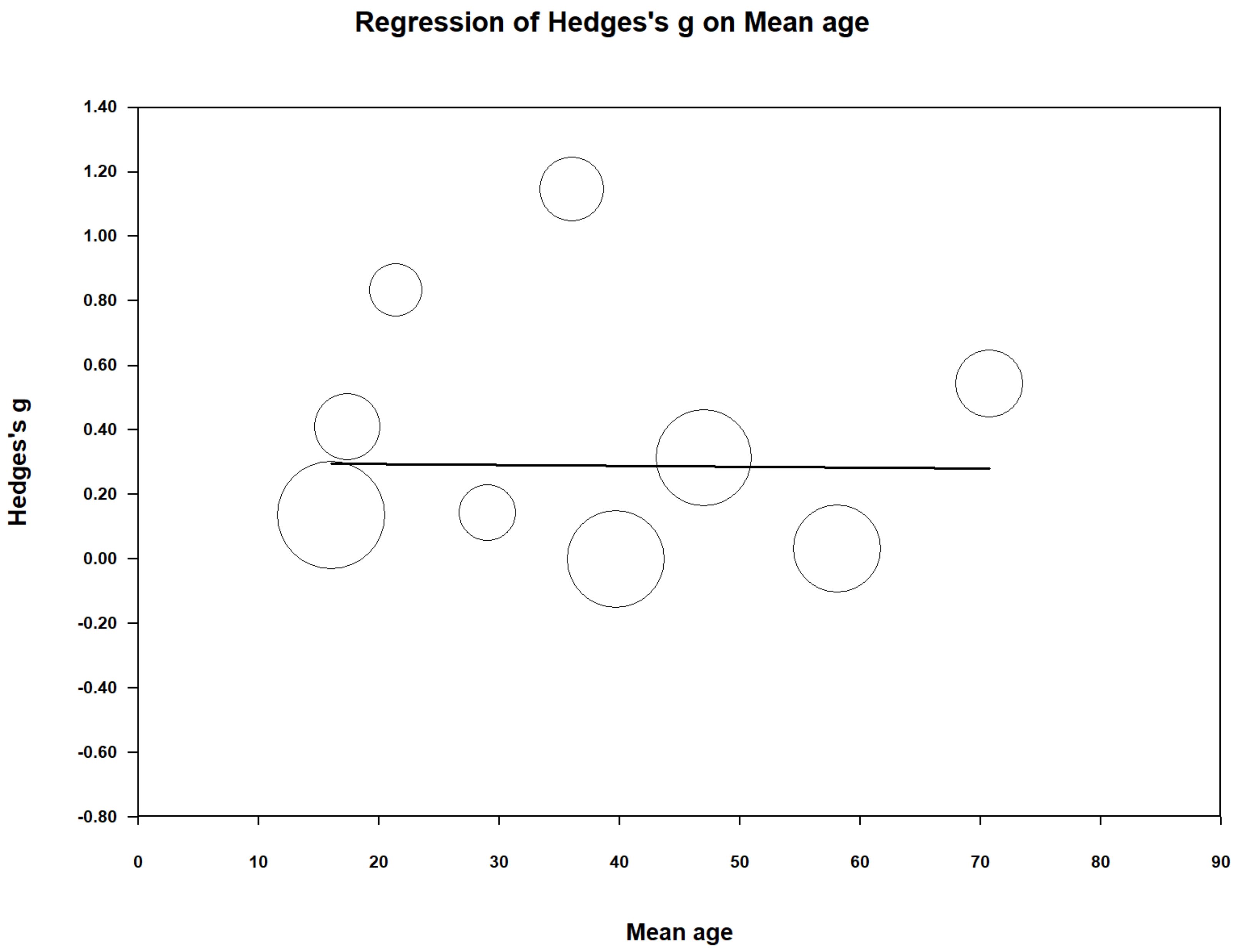

Figure S10. Meta-regression analysis examining the association between clubhead speed and mean participant age

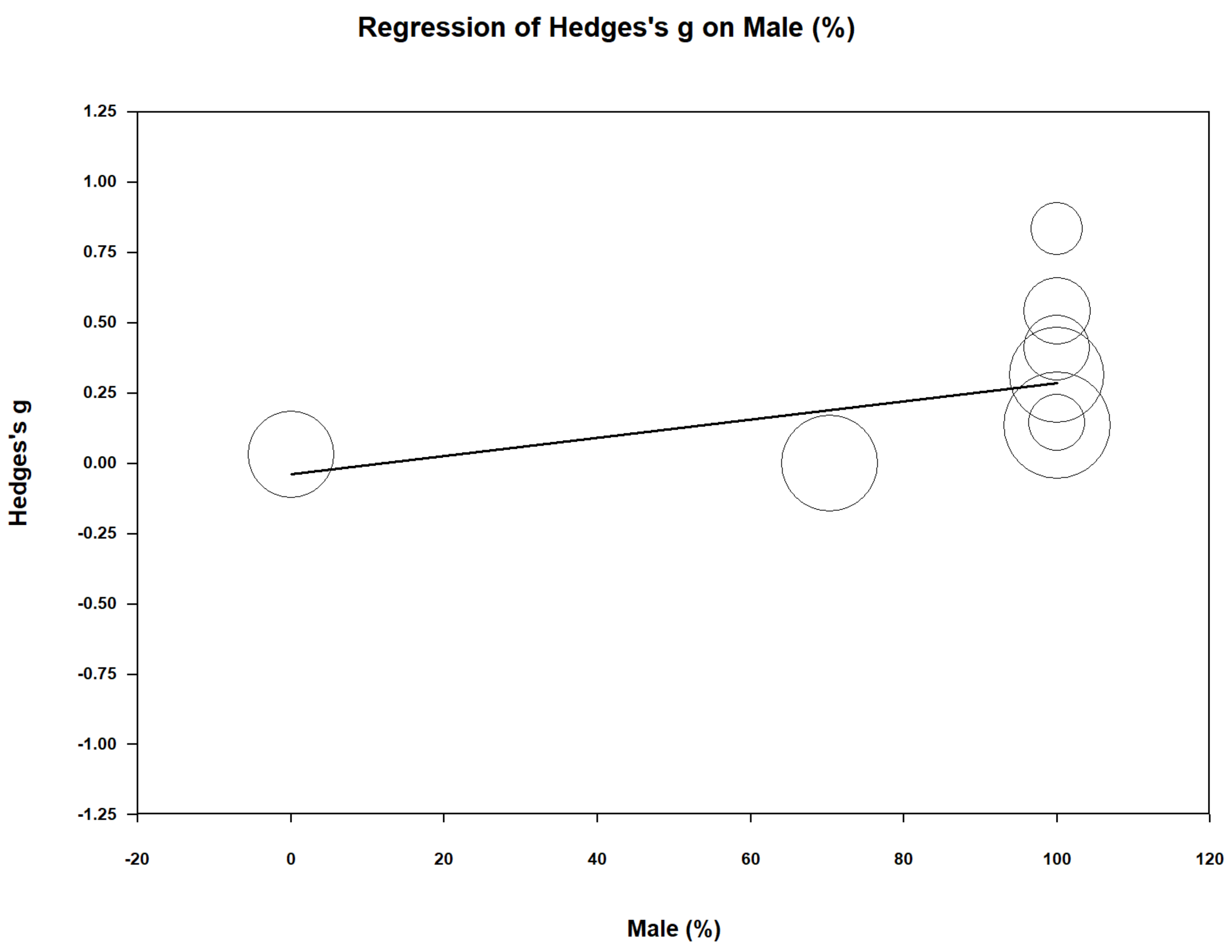

Figure S11. Meta-regression analysis examining the association between clubhead speed and proportion of male participants

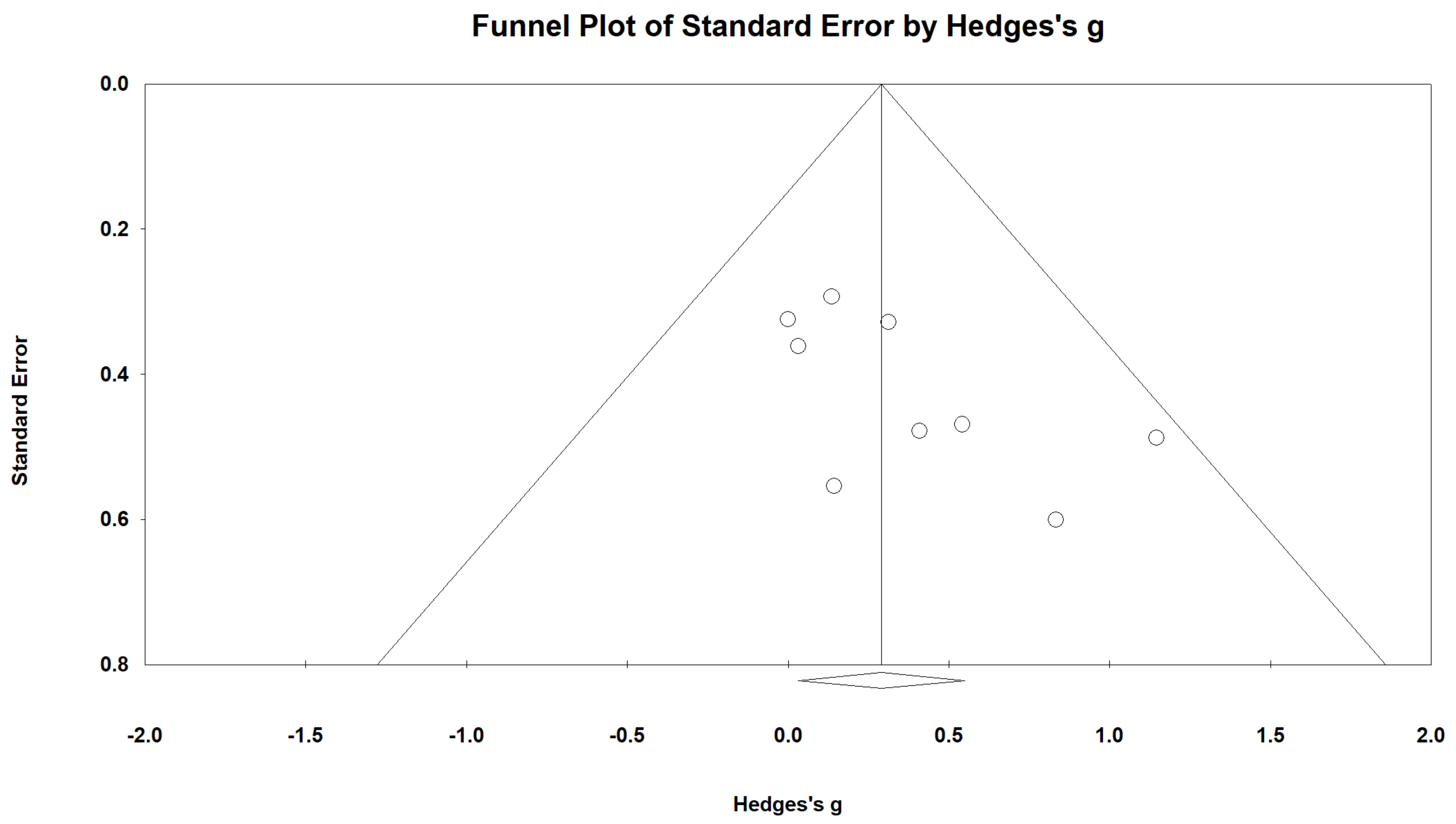

Figure S12. Funnel plot of the studies evaluating clubhead speed for publication bias
